# Active-site mTOR inhibitors augment HSV1-dICP0 infection in cancer cells via dysregulated eIF4E/4E-BP axis

**DOI:** 10.1371/journal.ppat.1007264

**Published:** 2018-08-23

**Authors:** Chadi Zakaria, Polen Sean, Huy-Dung Hoang, Louis-Phillipe Leroux, Margaret Watson, Samuel Tekeste Workenhe, Jaclyn Hearnden, Dana Pearl, Vinh Tai Truong, Nathaniel Robichaud, Akiko Yanagiya, Soroush Tahmasebi, Seyed Mehdi Jafarnejad, Jian-Jun Jia, Adrian Pelin, Jean-Simon Diallo, Fabrice Le Boeuf, John Cameron Bell, Karen Louise Mossman, Tyson Ernst Graber, Maritza Jaramillo, Nahum Sonenberg, Tommy Alain

**Affiliations:** 1 Goodman Cancer Centre, Department of Biochemistry, McGill University, Montreal, Canada; 2 Children's Hospital of Eastern Ontario Research Institute, Department of Biochemistry, Microbiology and Immunology, University of Ottawa, Ottawa, Ontario, Canada; 3 INRS Institut Armand-Frappier Research Centre, Laval, Quebec, Canada; 4 Department of Pathology and Molecular Medicine, MG DeGroote Institute for Infectious Disease, McMaster University, Hamilton, Ontario, Canada; 5 Center for Innovative Cancer Research, Ottawa Hospital Research Institute, Ottawa, Ontario, Canada; University of North Carolina at Chapel Hill, UNITED STATES

## Abstract

Herpes Simplex Virus 1 (HSV1) is amongst the most clinically advanced oncolytic virus platforms. However, efficient and sustained viral replication within tumours is limiting. Rapamycin can stimulate HSV1 replication in cancer cells, but active-site dual mTORC1 and mTORC2 (mammalian target of rapamycin complex 1 and 2) inhibitors (asTORi) were shown to suppress the virus in normal cells. Surprisingly, using the infected cell protein 0 (ICP0)-deleted HSV1 (HSV1-dICP0), we found that asTORi markedly augment infection in cancer cells and a mouse mammary cancer xenograft. Mechanistically, asTORi repressed mRNA translation in normal cells, resulting in defective antiviral response but also inhibition of HSV1-dICP0 replication. asTORi also reduced antiviral response in cancer cells, however in contrast to normal cells, transformed cells and cells transduced to elevate the expression of eukaryotic initiation factor 4E (eIF4E) or to silence the repressors eIF4E binding proteins (4E-BPs), selectively maintained HSV1-dICP0 protein synthesis during asTORi treatment, ultimately supporting increased viral replication. Our data show that altered eIF4E/4E-BPs expression can act to promote HSV1-dICP0 infection under prolonged mTOR inhibition. Thus, pharmacoviral combination of asTORi and HSV1 can target cancer cells displaying dysregulated eIF4E/4E-BPs axis.

## Author summary

Dysregulated mRNA translation occurs frequently in tumours due to elevated eIF4E expression or a hyperactive mTOR complex 1 (mTORC1) signaling pathway that results in the inactivation of the eIF4E binding proteins (4E-BPs). Targeting the mTORC1/4E-BPs/eIF4E axis is a promising strategy in cancer therapies and for preventing resistance to treatment. Enhanced mTORC1 activity also drives innate immune responses by modulating protein expression of antiviral genes. It was previously shown that the mTORC1 inhibitor rapamycin limits antiviral responses and promotes replication of oncolytic viruses within tumour tissues. Active-site dual mTORC1 and mTORC2 inhibitors (asTORi) have been developed for superior mTOR inhibition and anti-cancer potency but have not been studied in the context of oncolytic viral infection. We show here that prolonged treatment with asTORi strongly augments infection of HSV1-dICP0 in cancer cells, but not in normal cells, an effect modulated via eIF4E/4E-BP expression. Thus, cancer cells with dysregulated translation could be amenable to the pharmacoviral combination of HSV1 and asTORi treatment.

## Introduction

Oncolytic viruses are promising immunotherapeutic agents for the treatment of cancer [1]. HSV1 application is amongst the most advanced and successful oncolytic platforms; with Amgen’s oncolytic HSV1 talimogene laherparepvec (T-Vec, Imlygic) being the first oncolytic virus to receive FDA and EMA (Food and Drug Administration and European Medicines Agency) approval in October 2015 [2]. Numerous groups are developing oncolytic HSV1 variants, including the pre-clinical development of an infected-cell-protein-0-deleted HSV1 (HSV1-dICP0) [3]. Rapid viral clearance within tumour tissues constitutes a limitation for oncolytic viral therapies. Thus, potentiating viral replication could further increase efficacy [4].

We, and others reported that the drug rapamycin, an allosteric inhibitor of mTORC1, and approved therapy for certain cancers, improves oncolytic viral replication within tumours through suppression of innate immunity and type-I IFN production [5–7]. The protein kinase mTOR, which integrates extra- and intracellular signals to affect cellular growth, proliferation, metabolism, and survival, exists in two complexes: mTORC1, which is sensitive to rapamycin and regulates mRNA translation, and mTORC2, which primarily controls actin cytoskeleton organization [8]. Evidence for the role of mTORC1 signaling in innate immunity emerged from the findings that rapamycin suppresses type-I IFN in plasmacytoid dendritic cells (pDCs), which are the major producers of systemic type-I IFN [9]. Thereafter, it was shown that silencing or chemically inhibiting upstream activators of mTORC1 compromises innate immunity [5, 9–11], and that genetic deletion of the mTORC1 downstream targets ribosomal S6 kinases (S6Ks) results in impaired type-I IFN response [5, 12]. Conversely, depletion of the upstream mTORC1 repressors TSC1/2, or the absence of the translational repressors eIF4E-binding proteins 1 and 2 (4E-BP1/2) downstream of mTORC1, leads to enhanced interferon-regulatory factor 7 (*Irf-7*) mRNA translation and type I IFN production [13–16]. These studies have highlighted the mTOR signaling pathway as a critical component of innate immunity by controlling translation initiation of antiviral mRNAs [17].

Initiation of protein synthesis generally involves the recognition of the mRNA 5’-m^7^G-cap structure by the eIF4F complex; consisting of eIF4E, a cap-binding protein; eIF4A, an RNA helicase and eIF4G, a scaffolding protein that recruits eIF3 and the 40S ribosomal subunit to the mRNA [18]. eIF4E can be sequestered from eIF4F by 4E-BPs; in conditions of mTORC1 inhibition, hypophosphorylated (activated) 4E-BP1/2, and inducible 4E-BP3, bind strongly to eIF4E and repress translation, whereas hyperphosphorylated (inactivated) 4E-BPs do not, allowing formation of the eIF4F complex on the mRNA and initiation of protein synthesis [19, 20]. In cancer, mRNA translation is frequently dysregulated because of increased eIF4E protein levels and/or elevated phosphorylation of 4E-BPs due to a hyperactivated mTORC1 pathway [21]. Targeting the aberrant mRNA translation in cancer represents an attractive strategy; while the allosteric mTORC1 inhibitor rapamycin is a poor activator of 4E-BPs, ATP-competitive active-site mTORC1 and 2 inhibitors (asTORi), are superior to rapamycin in inhibiting mTORC1/2, activating 4E-BPs, and providing potent anti-cancer effects [22]. Interestingly, the ratio of eIF4E to 4E-BPs was shown to determine the anti-proliferative efficacy of asTORi [23, 24].

In contrast to rapamycin, which was previously reported to increase HSV1 infection in some cancer cells [7], asTORi treatment strongly limits HSV1 replication in normal cells [25, 26]. Strikingly, while suppressing viral replication in normal fibroblasts and epithelial cells, we observed that prolonged exposure to asTORi dramatically increases HSV1-dICP0 infection in cancer cells. asTORi treatment results in reduction of type-I IFN responses in both cancer and non-transformed cell lines, and strongly inhibits viral mRNA translation in normal cells. In contrast, viral protein synthesis persists in cancer cells and cells transduced to either increase eIF4E levels or deplete 4E-BP1/2, ultimately resulting in enhanced viral replication and spread. Importantly, the combination of asTORi and HSV1-dICP0 reduces tumour size of an aggressive syngeneic breast cancer mouse model. Thus, our data reveal that cancer cells harboring altered mRNA translation via dysregulated eIF4E/4E-BPs axis can be targeted by the combination of asTORi and HSV1-dICP0.

## Results

### asTORi treatment enhances HSV1 infection of cancer cells, but suppresses infection in non-transformed cells

**Fig 1:**
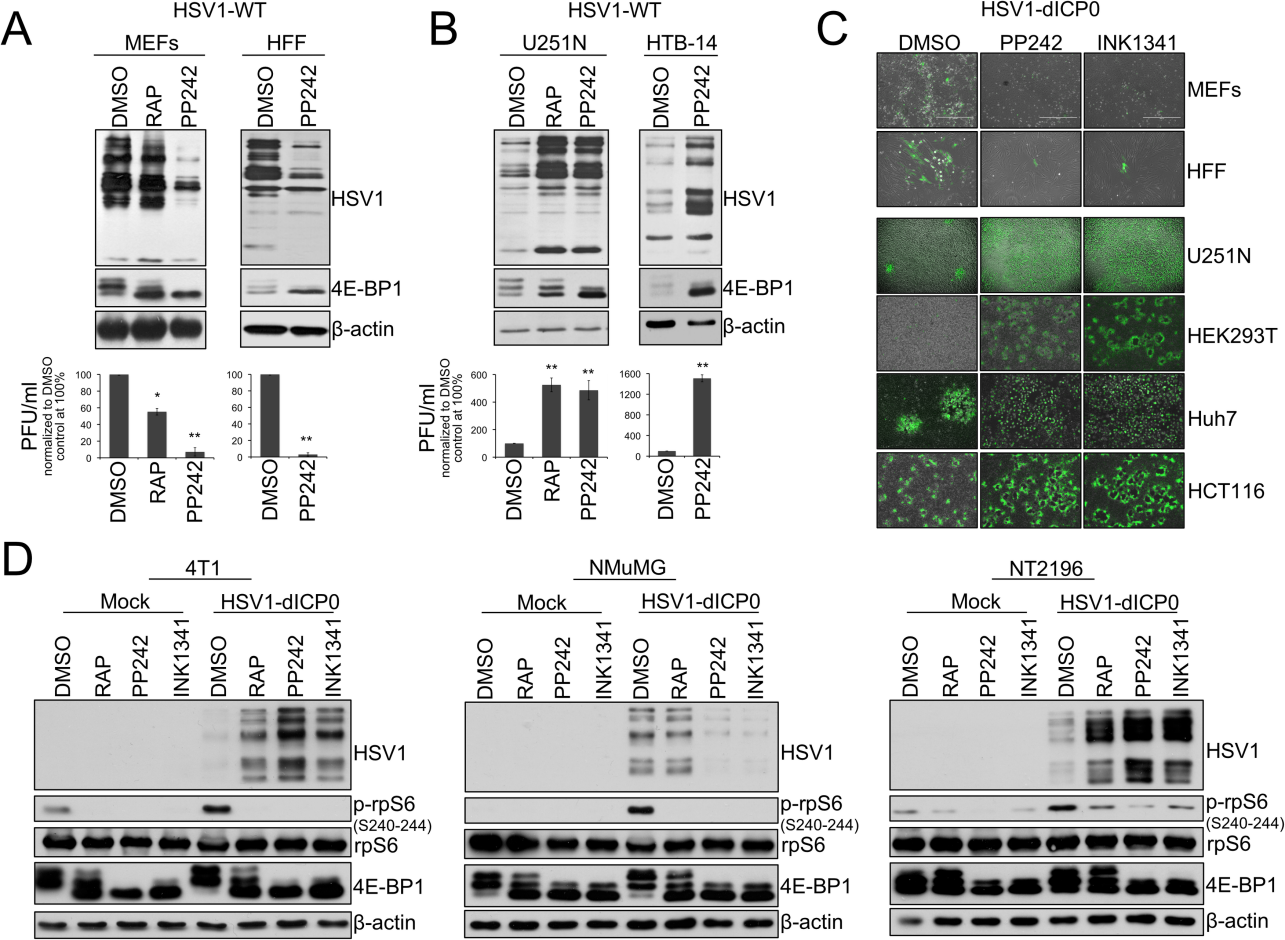
Active-site mTOR inhibitors (asTORi) augment HSV1 infection specifically in transformed cells. (A) Primary mouse embryonic fibroblasts (MEFs), human foreskin fibroblasts (HFF), and (B) human glioblastoma cell lines U251N and HTB-14 were pretreated with DMSO, rapamycin (RAP 100nM), or the asTORi PP242 (2μM) for 30min followed by infection with wild type HSV1 at a MOI of 0.1 for 48 hours in presence of the inhibitors. Viral infection was monitored by Western blot using antibodies against HSV1 antigens (top panel - 4E-BP1 and β-actin expression were used to monitor drug efficacy and loading, respectively), and plaque titration (bottom panel—results are presented as titers normalized to DMSO control set at 100% ± SD (n = 3)). (C) Non-transformed MEFs and HFF, as well as different transformed human cell lines, were infected with GFP-expressing HSV1-dICP0 for 48 hours at a MOI of 0.1. Resulting infection was assessed by fluorescence microscopy. (D) Transformed (4T1 and NT2196) and non-transformed (NMuMG) mouse mammary cell lines were pretreated with DMSO, rapamycin (100nM), PP242 (2μM) or INK1341 (100nM) for 30 min followed by infection with a GFP-expressing HSV1-dICP0 (0.1 MOI for 48 hours in presence of the inhibitors). Viral protein synthesis was monitored by Western blot against HSV1. Drug efficacy was monitored by phosphorylation of rpS6 and 4E-BP1. Total rpS6 and β-actin expression were used as loading controls.

Rapamycin augments the oncolytic potential of several viruses [5–7, 27–29]. asTORi are currently in clinical development [22, 30]. Therefore, we sought to determine whether the asTORi PP242 and INK1341 could augment the infection of different oncolytic viruses in cancer cells. The human glioblastoma cell line U251N or the mouse mammary carcinoma cell line 4T1 were infected with several oncolytic viruses including vesicular stomatitis virus (GFP-expressing VSV^Δ51M^), myxoma virus (GFP-expressing MV), vaccinia virus (GFP-expressing JX594) and HSV1 (infected cell protein 0 (*ICP0*)-defective oncolytic HSV1 expressing GFP (HSV1-dICP0)) in the presence or absence of asTORi. Our data showed that asTORi treatment limited VSV^Δ51M^, MV, and vaccinia virus infection, but unpredictably strongly increased HSV1-dICP0 infection and spread by 48 hours post-treatment **(S1A–S1D Fig**). This was unexpected as it had been previously reported that asTORi strongly suppress wild type HSV1 infection of primary human fibroblasts and primary mouse embryonic fibroblasts (MEFs) [25, 26]. To address this conundrum, we infected primary human foreskin fibroblasts (HFF) and MEFs with wild type HSV1 in the presence of rapamycin, or the asTORi PP242. As reported, wild type HSV1 infection was repressed (more than 10-fold) by treatment with PP242 in normal cells (**Fig 1A**). In stark contrast, this treatment markedly enhanced (5–10 fold) wild type HSV1 infection in the human glioblastoma cell lines U251N and HTB-14 (**Fig 1B**). Similar to asTORi, rapamycin enhanced HSV1 infection in U251N (**Fig 1B**) and 4T1 cells (**S1D Fig**), however unlike asTORi, rapamycin had only a partial effect in suppressing virus expression in MEFs (**Fig 1A**).

**Fig 2:**
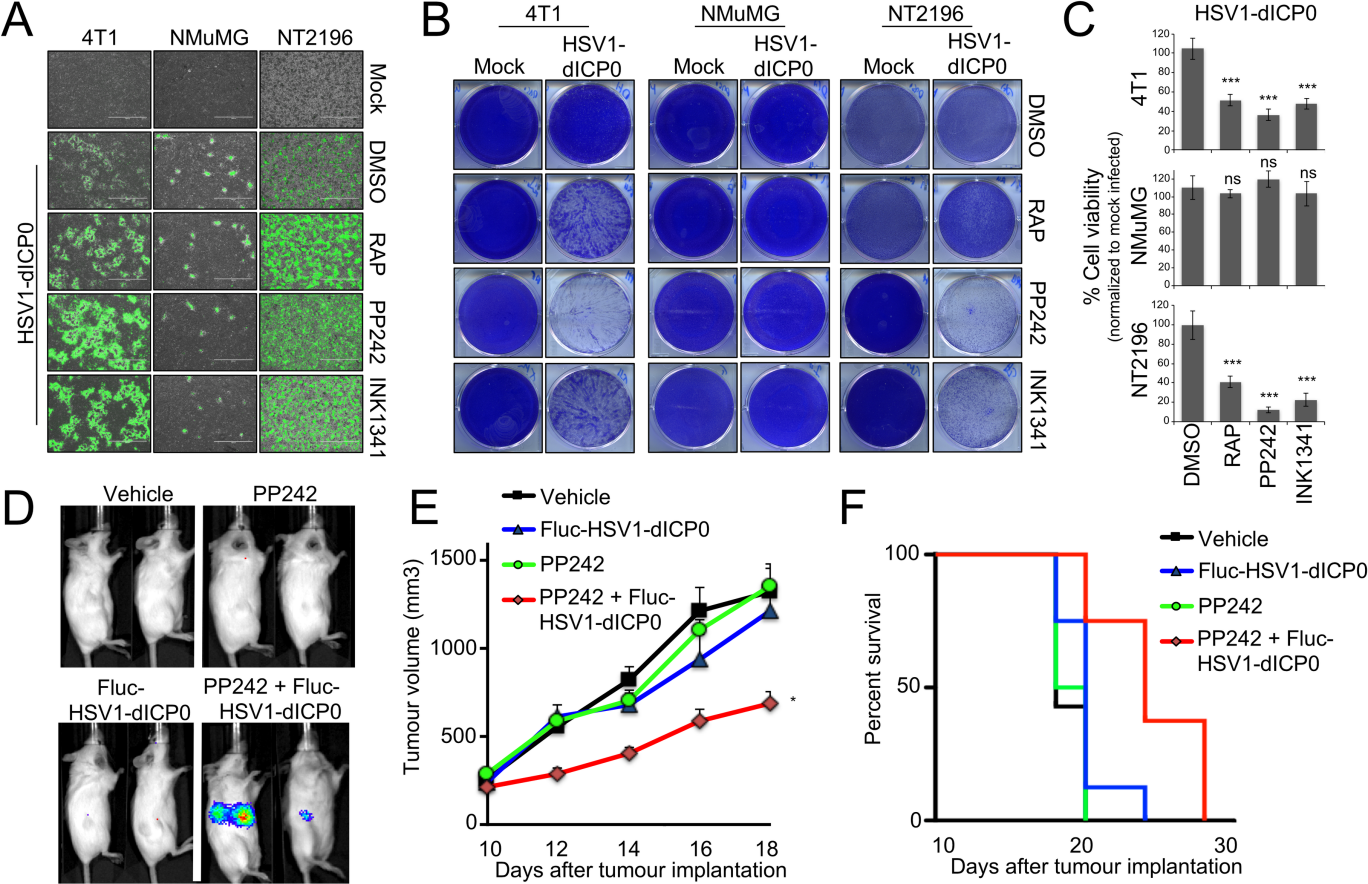
asTORi specifically enhance HSV1-dICP0 oncolysis in transformed cells and tumours. Mouse mammary carcinoma cell lines (4T1 and NT2196) and normal mammary epithelial cells (NMuMG) were pretreated with DMSO, rapamycin (RAP 100nM), PP242 (2μM) or INK1341 (100nM) for 30min followed by infection with GFP-expressing HSV1-dICP0 (0.1 MOI) in the presence of inhibitors. At 48 hours post-infection, cell infection was monitored by (A) fluorescence microscopy, and at 96 hours post-infection, viability and oncolysis were monitored by (B) crystal violet staining of live cells, and (C) trypan blue exclusion (represented as mean number of viable cells ± SD normalized to respective mock infected control (n = 3)). (D-F) Luciferase-expressing HSV1-dICP0 infection is restricted to tumour bearing mice treated with PP242. 4T1 cells were implanted subcutaneously in the right flank of syngeneic mice. Once tumours were palpable (10 days post-implantation), mice were treated with PP242 (60mg/kg) or vehicle by gavage (days 10,12,14,17 and 18), and injected or not with 10^7^ pfu of FLuc-HSV1-dICP0 intra-tumourally (days 11 and 13) (n = 5 in each group). Luciferase expression was measured by IVIS on day 14 (D). Size of tumours was monitored by electronic caliper (E), and overall survival was determined at end point (F).

We then examined the infection of various cancer cell lines with HSV1-dICP0 [3]. Supporting our data in U251N and 4T1 cells, asTORi (PP242, INK1341, INK128 or Torin1) treatment increased HSV1-dICP0 infection and viral protein levels in multiple transformed human cell lines (HEK293T, HCT116, Huh7) and mouse mammary tumour cell lines (4T1, NT2196) by 48–72 hours post-infection. In contrast, viral infection and protein synthesis were repressed in several cell lines derived from normal tissues that were treated with asTORi: fibroblasts (MEF, HFF), the non-transformed neuroblast cell line SHEP [31, 32], and the mouse epithelial mammary cell line NMuMG (**Fig 1C and 1D, S1E and S1F Fig and S2A–S2D Fig**). To better understand the kinetics of the enhanced viral infection in the presence of the asTORi PP242, we used IncuCyte real-time imaging to monitor GFP-expressing HSV1-dICP0 in both the NMuMG cell line and the NeuT-transformed NMuMG cell line NT2196 [33]. We found that the increased GFP expression in the transformed NT2196 cells relative to DMSO-treated cells begins only at 24 hours post-infection, and that asTORi strongly suppressed the virus in the normal NMuMG cells (**S1E Fig**). Importantly, this effect was not unique to HSV1-dICP0, as g34.5-deleted HSV1-1716, that has undergone clinical development [34], also led to an increase in fluorescence upon treatment with PP242 as compared to DMSO control in the transformed NT2196 cell line (**S1E Fig**). However, the increase with g34.5-deleted HSV1-1716 was not as dramatic as seen with HSV1-dICP0. Furthermore, g34.5-deleted HSV1-1716 led to no detectable GFP fluorescence in the normal breast epithelial cell line NMuMG. Therefore, to characterize the differences between normal and transformed cells, we pursued experiments primarily using HSV1-dICP0. By comparing 4T1 and NT2196 cells to NMuMG, we confirmed that combining asTORi with HSV1-dICP0 potentiates viral infection (measured by Western blot, GFP expression, and titration), and oncolysis (measured by crystal violet and trypan blue exclusion) in the transformed mammary cells (**Fig 1D, Fig 2A–2C, S1E and S1F Fig and S2E Fig**). Furthermore, intratumoural injection of luciferase-expressing HSV1-dICP0 in mice bearing late-stage 4T1 tumours showed detectable luciferase expression only in tumours of mice that had been administered asTORi (**Fig 2D**). Correspondingly, the combinatorial pharmacoviral treatment resulted in significant reduced tumour growth (by ~50% at day 18 post-implantation, p = 0.019 and p = 0.021 over PP242 or HSV1-dICP0 monotherapy respectively), and moderately prolonged animal survival bearing this aggressive breast tumour as compared to either pharmacological or viral single therapy (median survival 15 days post-treatment for the combination compared to 8–10 days for PP242 or HSV1-dICP0 monotherapies, respectively, p = 0.045) (**Fig 2E and 2F**). Collectively, these data demonstrate that mTOR inhibition by asTORi suppresses HSV1 replication in non-transformed fibroblasts and epithelial cells (as previously reported [25, 26], but surprisingly the same treatment causes a robust, albeit delayed, enhancement of HSV1 replication in cancer and transformed cells.

### asTORi treatment reduces cellular type-I IFN responses

**Fig 3:**
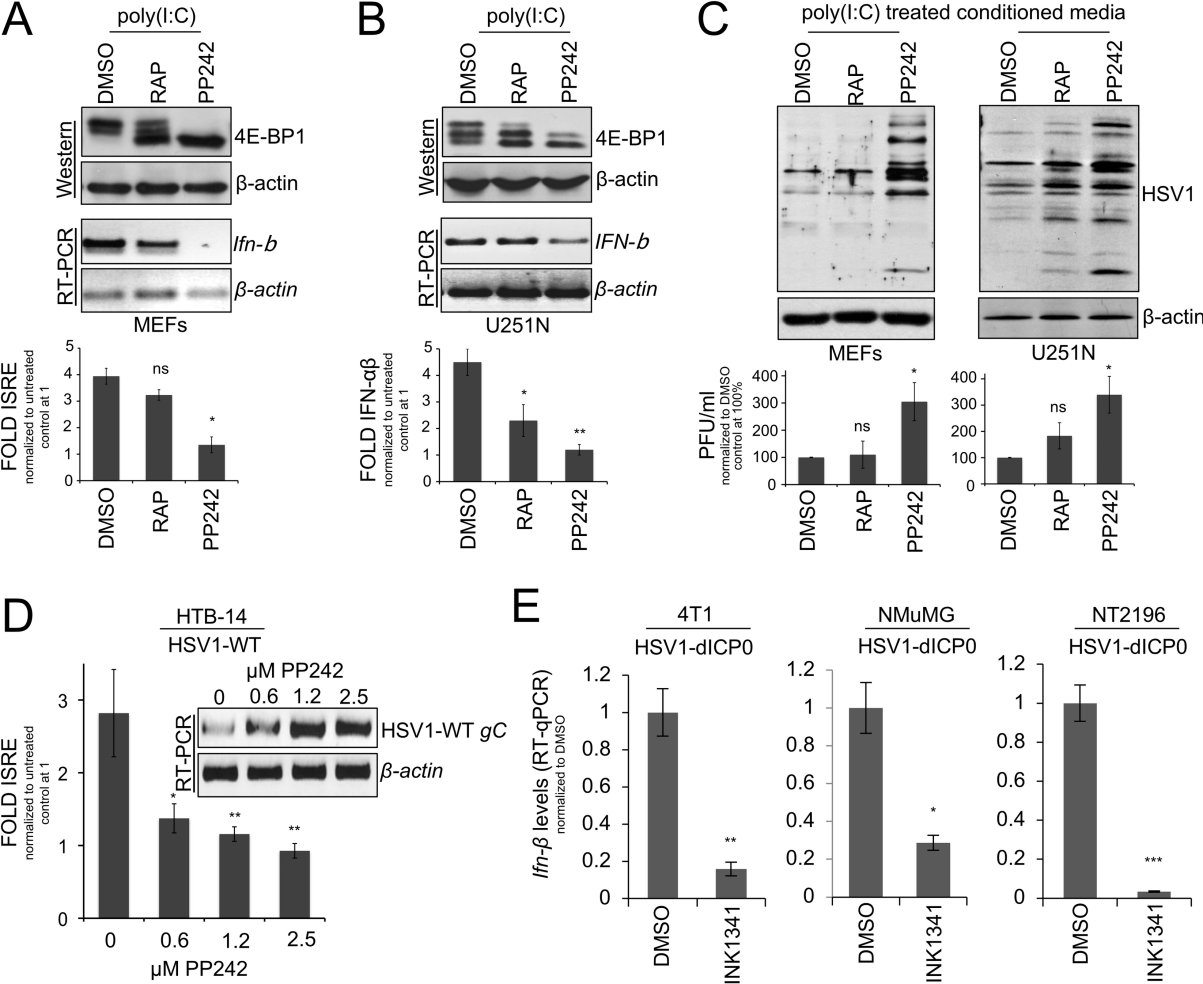
asTORi suppress cellular type-I IFN responses. Non-transformed mouse embryonic fibroblasts (MEFs) (A), or the human glioma cell line U251N (B) were transfected with 1μg/ml poly(I:C) RNA in the presence of DMSO, rapamycin (RAP 100nM) or PP242 (2μM). mTORC1 inhibition was monitored by assessing the phosphorylation status of 4E-BP1 by Western blot (A and B top). *Ifn-β* transcript levels were measured by RT-PCR (A and B middle), and type-I IFN responses were measured using ISRE reporter activity assay (A bottom), or HEK-Blue type-I IFN assay (B bottom), presented as fold change compared to untreated control cells set to 1 ± SD (n = 3). (C) Protection assay, MEFs or U251N cells were transfected with 1μg/ml poly(I:C) RNA and treated as in (A,B). The supernatant collected 24 hours later was used to condition “naïve” MEFs or U251N cells for 6 hours followed by wild type HSV1 infection for 24 hours. Viral replication in the conditioned cells was monitored by Western blot (C top) and plaque assay titration (C bottom–results are presented as titers normalized to DMSO control set at 100% ± SD (n = 3)). (D) HTB-14 cells were transfected with an ISRE reporter and infected with wild type HSV1 at 1 MOI in the presence of DMSO or increasing concentrations of PP242. Concomitant luciferase counts of ISRE reported activity (presented as fold change over mock control set to 1 ± SD (n = 3)) and wild type HSV1 *gC* transcripts levels were monitored by luciferase assays and by RT-PCR, respectively. (E) Transformed 4T1 and NT2196 cells, as well as non-transformed NMuMG cells, were treated with DMSO or INK1341 (100μM) and infected with GFP-expressing HSV1-dICP0 (0.1 MOI) for 24 hours. *Ifn-β* mRNA levels were measured by RT-qPCR. Results are presented as fold change normalized to DMSO control set to 1 ± SD (n = 3).

Inhibition of mTORC1 by rapamycin limits type-I IFN responses [5, 6, 9]. Additionally, mTORC2 has been implicated in the regulation of innate immunity [35]. Thus, asTORi that strongly impede the activation of both mTORC1 and 2, and potently activate 4E-BPs, are expected to exhibit robust inhibition of type-I IFN production and antiviral responses in cells. To assess whether asTORi treatment and activation (hypophosphorylation) of 4E-BPs limits type-I IFN signaling, we transfected synthetic double stranded poly(I:C) RNA to mimic natural infection by viruses, or infected primary MEFs and the glioma U251N cells with wild type HSV1 in the presence of rapamycin or the asTORi PP242. As previously reported [36, 37] and shown above (**Fig 1A and 1B**), asTORi treatment resulted in complete dephosphorylation of 4E-BP1 in both MEFs and U251N cells, whereas rapamycin exerted only a partial effect on 4E-BP1 phosphorylation (**Fig 3A and 3B**, top panels). RT-PCR for *Ifn-β* after poly(I:C) RNA transfection (**Fig 3A and 3B**, middle panels), or wild type HSV1 infection (**S3A Fig**), revealed that cells treated with asTORi had limited induction of *Ifn-β* mRNA expression as compared to rapamycin or DMSO control. Similarly, the ~4–5 fold increase in interferon-stimulated response element (ISRE) promoter activity and type-I IFN production in presence of poly(I:C) RNA was blocked in PP242-treated cells while a partial reduction was detected in rapamycin-treated cells (**Fig 3A and 3B**, bottom panels). Using conditioned media from MEFs or U251N cells treated with poly(I:C) RNA and DMSO (control), rapamycin, or asTORi, a reduced protection from subsequent wild type HSV1 infection was primarily observed in asTORi treated cells (**Fig 3C and S3B Fig**). Similarly, RT-PCR for HSV1 *gC* transcripts and ISRE reporter activity from the human glioblastoma HTB-14 cells infected with wild type HSV1, confirmed that PP242 treatment limits the induction of ISRE reporter activity, with a corresponding increase in HSV1 *gC* transcript levels (**Fig 3D**). Furthermore, the induction of *Ifn-β* mRNA levels measured by RT-qPCR was suppressed to a similar extent upon asTORi treatment and HSV1-dICP0 infection in 4T1, NT2196, and NMuMG cells (**Fig 3E**). Additionally, the inhibitory effect of asTORi on type I IFN production was maintained in poly(I:C)-stimulated normal human foreskin fibroblasts HFF and human glioblastoma cell lines U343 and U373 (**S3C Fig**). To further demonstrate that dual mTORC1/2 inhibitors impair the innate immune response, we performed polysome profiling and translation reporter assays of cellular and viral mRNAs. These experiments showed that *Irf7* mRNA is excluded from polysomes in 4T1 and NT2196 cells infected with HSV1-dICP0 in the presence of the asTORi PP242 (**S3D–S3G Fig**). In contrast, the interferon-induced gene *Isg15* was rather weakly induced transcriptionally upon asTORi treatment as compared to control (**S3G Fig**). Importantly, these experiments also demonstrated that HSV1-dICP0 viral mRNAs (*ICP4*, *gC*, *TK*) are highly transcribed and more abundant in polysome fractions when 4T1 or NT2196 cancer cells were treated with asTORi as compared to DMSO control (**S3E–S3G Fig**). To examine the translational repression of *Irf7* mRNA versus HSV1 genes, we generated reporter constructs by merging the 5’ UTR of *Irf7*, or those of HSV1-*TK* or *ICP0*, to a luciferase reporter gene, and measured luciferase expression upon DMSO (control) versus asTORi treatment in 4T1 cells. *TK* and *ICP0* were selected since they have well annotated 5’ UTR that could be readily cloned into our reporter system. As expected from previous data using the asTORi Torin1 or silencing of eIF4E [38], these experiments showed that the reporter construct containing the 5’ UTR of *Irf7*, which is highly structured [14], was repressed translationally, in contrast to the reporter constructs containing the *ICP0* or the *TK* 5’ UTR (**S3H Fig**). These results demonstrate that mTORC1/2 inhibition by asTORi strongly limits innate antiviral responses and production of type-I IFN in both normal and cancer cells.

### asTORi treatment represses host and viral protein synthesis in normal cells, but viral mRNA translation persists in cancer cells

**Fig 4:**
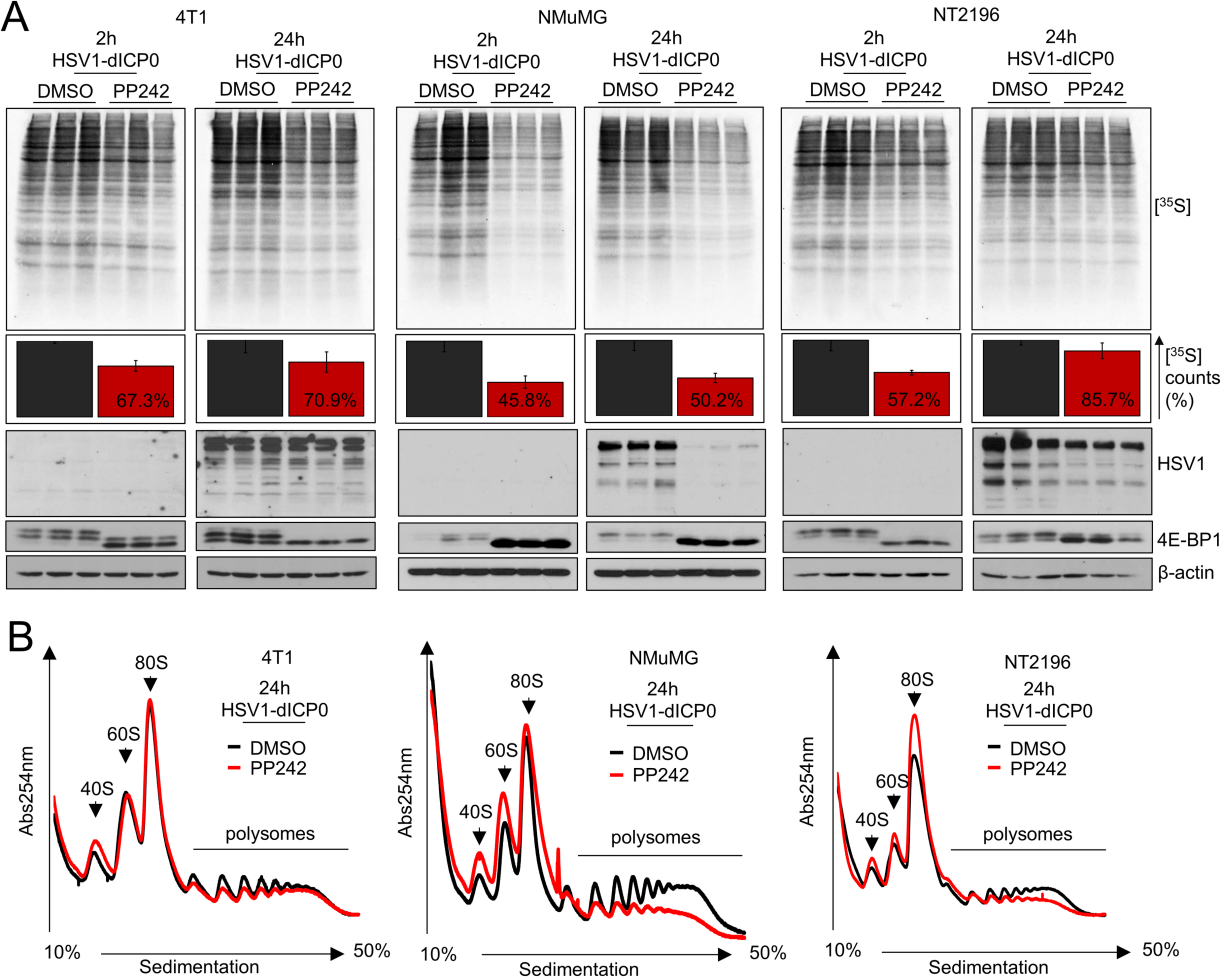
The asTORi PP242 suppresses host and viral protein synthesis in normal cells but sustains HSV1-dICP0 protein synthesis in cancer cells. (A) 4T1, NMuMG and NT2196 cells were infected in triplicates with GFP-expressing HSV1-dICP0 (0.1 MOI) in the presence of DMSO or PP242 (2μM), pretreated for 30 min prior to infection. Global protein synthesis was assessed by a 30 min pulse [^35^S]methionine incorporation into newly synthesized proteins at 2 hours and 24 hours post-infection. Triplicate cell lysates were separated on SDS-PAGE and changes in protein synthesis were revealed by autoradiography (top), [^35^S]methionine incorporation was measured by TCA precipitation and quantified on scintillation counter (middle–results presented are normalized to DMSO control set to 100% ± SD (n = 3)), and 4E-BP1 phosphorylation status as well as the levels of HSV1 proteins were assessed by Western blot (bottom). (B) Polysome profile analysis of 4T1, NMuMG and NT2196 cells treated and infected with HSV1-dICP0 as above for 24 hours.

Our results show that asTORi treatment suppresses type-I IFN signaling in both normal fibroblast/epithelial cells and cancer cell lines. To determine whether early infection of HSV1-dICP0 was accelerated by asTORi treatment, we assessed the mRNA levels of the immediate early viral gene ICP4 at 8 hours post-infection in 4T1, NMuMG and NT2196 cells treated either with DMSO or the asTORi INK1341. RT-qPCR data revealed no significant changes in *ICP4* mRNA levels between the conditions used for all cell lines, suggesting that viral entry was generally not affected by asTORi treatment (**S4A Fig**). Therefore, we reasoned that the specific augmentation of HSV1-dICP0 replication in cancer cells cannot be explained by either a more pronounced reduction in type-I IFN responses, or an increase in viral entry. Protein synthesis is frequently dysregulated in cancer and contributes to resistance to therapeutic drugs, particularly mTOR inhibitors, by allowing the continued translation of a subset of mRNAs involved in cell proliferation [23, 24, 37]. Interestingly, a recent report demonstrated that transient fasting, a condition that causes reduced mTORC1 activity, enhances the replication of oncolytic HSV1 in glioblastoma cells, which was proposed to be mediated by dysregulated protein synthesis in cancer cells [39]. We examined global protein synthesis by supplementing with [^35^S]-Met in the growth media of 4T1, NT2196 and NMuMG cells infected with HSV1-dICP0 and treated with the asTORi PP242. PP242 and HSV1-dICP0 treatment resulted in reduction of global protein synthesis, an effect that was more pronounced and sustained in NMuMG cells as compared to the transformed 4T1 and NT2196 cell lines (~50% [^35^S]-Met incorporation over DMSO control for NMuMG, versus ~70% and 85% for 4T1 and NT2196, at 24 hours post-infection respectively) (**Fig 4A**). As previously observed for early time points post-infection in cancer cells (**S1A and S1E Fig**), HSV1-dICP0 protein levels at 24 hours post-infection were comparable in control (DMSO) and PP242-treated 4T1 and NT2196 cancer cells, but were barely detected in the normal epithelial cell line treated with PP242 (**Fig 4A**). In agreement with the [^35^S]-Met incorporation results, polysome profiling analyses showed that PP242 treatment engendered a stronger reduction in polysomal RNA in NMuMG cells than in 4T1 and NT2196 cell lines (**Fig 4B**). PP242 treatment also reduced [^35^S]-Met incorporation to a higher extent in HFF as compared to U251N, and suppressed wild type HSV1 protein expression only in HFF (**S4B Fig**). Interestingly, PP242 inhibited HSV1-dICP0 protein expression in the non-transformed NMuMG cells at low concentrations (0.4–0.6μM), while exerting the opposite effect on transformed NT2196 cells up 2μM. However, higher concentrations of asTORi ultimately reduced HSV1-dICP0 infection in certain cancer cell lines (**S4C and S4D Fig**), suggesting that excessive mTOR suppression can limit HSV1-dICP0 protein synthesis and concomitant infection. These data indicate that asTORi treatment induces a stronger and more sustained translational repression in normal cells than in cancer cells, which ultimately dampens efficient HSV1 protein synthesis and replication. Transformed cells maintain higher levels of protein synthesis under asTORi treatment, recover more quickly, and sustain HSV1 mRNA translation thereby promoting the replication of the virus.

### asTORi-dependent augmentation of HSV1-dICP0 propagation is altered by modulating eIF4E/4E-BP expression

**Fig 5:**
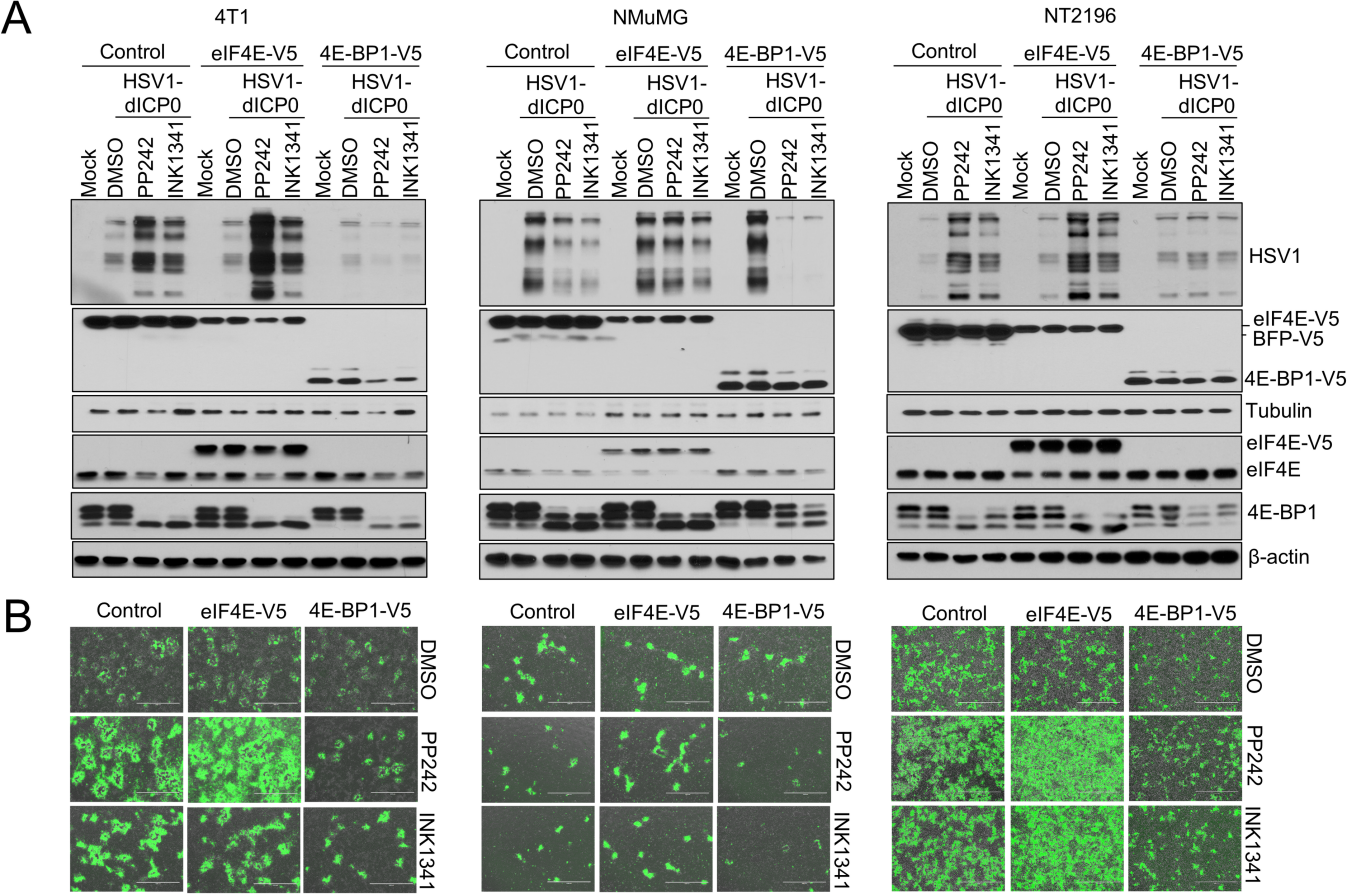
Overexpression of eIF4E or 4E-BP1 modulates the effects of asTORi treatment on HSV1-dICP0 infection. Transformed 4T1 and NT2196 and non-transformed NMuMG were transduced to stably overexpress V5-tagged eIF4E, 4E-BP1, or control (blue fluorescent protein—BFP). Transduced cells were infected with GFP-expressing HSV1-dICP0 (0.1 MOI) in the presence of DMSO, PP242 (2μM), or INK1341 (100nM), pretreated for 30 min prior to infection. At 48 hours post-infection, virus protein synthesis and infection were monitored by (A) Western blot and (B) fluorescence microscopy. Expression of V5-tag, eIF4E and 4E-BP1 showed drug efficacy and protein overexpression. β-actin was used as loading control.

The ratio of eIF4E/4E-BP protein expression determines the anti-proliferative efficacy of mTOR inhibitors [23, 24, 37, 40]. Elevated eIF4E expression, in relation to 4E-BP1/2/3, renders cells more resistant to the repression of proliferation and mRNA translation by asTORi. Interestingly, asTORi inhibition of wild type HSV1 in MEFs was previously shown to be dependent on 4E-BP1 expression [26]. Therefore, we hypothesized that dysregulated protein synthesis due to altered eIF4E/4E-BP ratio in cancer cells is responsible for the differences in HSV1-dICP0 infection between normal and cancer cells treated with asTORi. Initially, we sought to compare the ratio of eIF4E to 4E-BPs in normal and transformed mouse mammary cells lines. While eIF4E and 4E-BP1 protein expression appeared similar between these cells, the normal NMuMG cell line exhibited higher expression of 4E-BP2 (and a slight increase of 4E-BP3 upon mTOR inhibition) in comparison to the transformed 4T1 and NT2196 (**S4E Fig**). To test the hypothesis that elevated eIF4E or reduced 4E-BPs protein expression contributes to HSV1-dICP0 viral propagation in the presence of asTORi, we transduced 4T1, NT2196, and NMuMG cells with constructs expressing V5-tagged eIF4E, 4E-BP1 or the control Blue Fluorescent Protein (BFP). Strikingly, V5-4E-BP1 overexpression (i.e. reduced eIF4E/4E-BP1 ratio) prevented the asTORi-mediated increase of HSV1-dICP0 protein synthesis in transformed cells (4T1 and NT2196), and resulted in greater repression of HSV1-dICP0 infection by asTORi in normal cells (NMuMG). In contrast, eIF4E overexpression in the NMuMG cells led to sustained viral protein synthesis during asTORi treatment (**Fig 5A and 5B**).

**Fig 6:**
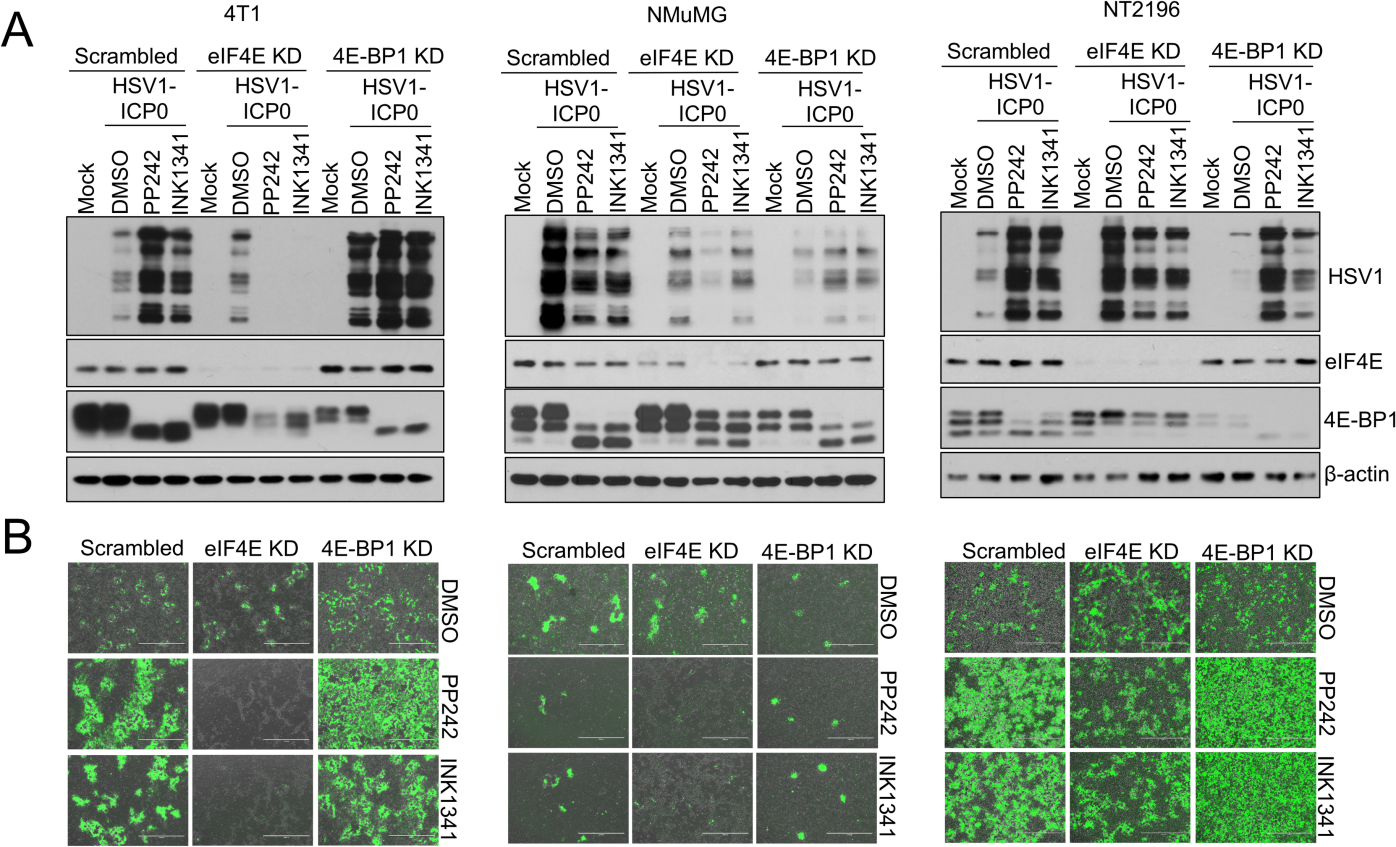
Silencing eIF4E or 4E-BP1 modulates the effects of asTORi treatment on HSV1-dICP0 infection. Transformed 4T1 and NT2196 and non-transformed NMuMG were transduced to stably express shRNA against eIF4E, 4E-BP1, or scrambled shRNA control. Transduced cells were infected with GFP-expressing HSV1-dICP0 (0.1 MOI) in the presence of DMSO, PP242 (2μM), or INK1341 (100nM), pretreated for 30 min prior to infection. At 48 hours post-infection, virus protein synthesis and infection were monitored by (A) Western blot and (B) fluorescence microscopy. Expression of eIF4E and 4E-BP1 showed drug efficacy and target knockdown. β-actin was used as loading control.

To confirm the contribution of the eIF4E/4E-BP axis in potentiating HSV1-dICP0 infection during asTORi treatment, we knocked down eIF4E, 4E-BP1 or 4E-BP2 in 4T1, NT2196 and NMuMG cells. Silencing eIF4E led to a strong repression of HSV1-dICP0 infection in cancer cells treated with asTORi, while silencing 4E-BP1 or 4E-BP2 had a reverse effect (**Fig 6A and 6B and S4F Fig**). To examine this response in other cell models, we used NIH3T3 mouse fibroblasts that overexpress eIF4E [41]. PP242 treatment in non-transformed control NIH3T3 cells limited wild type HSV1 infection. However, transformed NIH3T3 cells with excess levels of eIF4E were resistant to PP242-mediated suppression of wild type HSV1 protein expression (**S5A Fig**). Similar results were obtained using the non-transformed human neuroblast cell line SHEP [31, 32], where PP242 treatment decreased wild type HSV1 protein levels in cells treated with empty vector control, but not in cells overexpressing eIF4E (**S5B Fig**). As expected, a stronger suppression of wild type HSV1 protein expression was also observed in SHEP cells overexpressing 4E-BP1 (**S5C Fig**). Consistent with these findings, silencing eIF4E or increasing expression of 4E-BP1 in U251N cells diminished the elevated infection of HSV1-dICP0 in presence of asTORi, whereas eIF4E overexpression had the reverse effect (**S5D–S5H Fig**). These data demonstrate that cells with overexpression of eIF4E or reduced expression of 4E-BPs, can sustain HSV1 protein synthesis during mTOR inhibition. The results further support the notion that in cancer cells with dysregulated eIF4E/4E-BP ratio, administration of asTORi could promote the replication of HSV1-dCIP0.

## Discussion

In this study we investigated the pharmacoviral combination of asTORi and HSV1-dICP0. asTORi treatment suppressed type-I IFN responses in normal and cancer cells. The treatment impaired the replication of HSV1-dICP0 in non-transformed cells, but infection was strikingly augmented in cancer cells. Furthermore, in an aggressive syngeneic mammary cancer mouse model, the combination of asTORi and HSV1-dICP0 reduced tumour size and prolonged survival compared to either monotherapy. Mechanistically our data show that asTORi treatment initially represses global mRNA translation, but HSV1-dICP0 protein expression persists in cancer cells and cells with dysregulated eIF4E/4E-BP ratio. Since cancer cells with high eIF4E expression demonstrate resistance to the anti-proliferative effects of asTORi [23], combining dual mTORC1 and 2 inhibitors with HSV1-dICP0 could potentially provide the selective advantage of increasing viral oncolysis of mTOR-inhibitor resistant cancer cells.

The eIF4E/4E-BP1 ratio is a key determinant of cell response to mTOR inhibitors as tumour cells with a high eIF4E/4E-BP1 ratio exhibit low sensitivity to asTORi-induced cell proliferation arrest [23, 24, 37]. Furthermore, in hepatocellular carcinoma cells or aggressive B-cell lymphomas, high eIF4E/4E-BP1 confers resistance to metformin-induced apoptosis or mTOR inhibitors, respectively [24, 40]. In addition, the mTORC1 signaling pathway is often hyperactivated in cancer resulting in elevated phosphorylation of 4E-BPs [21]. mTORC1 activity was previously demonstrated to be required for wild type HSV1 infection and replication as primary human and mouse fibroblasts treated with asTORi were resistant to wild type HSV1 infection [25, 26]. Interestingly, Moorman and Shenk showed that primary MEFs depleted of 4E-BP1 produced equal yields of HSV1 in the presence or absence of asTORi [26]. We also report here that asTORi treatment potently repressed wild type HSV1 and HSV1-dICP0 in several normal cell lines, but found that viral replication and spread is markedly augmented under prolonged asTORi treatment within cancer cells. We further show that varying the cellular expression levels of eIF4E, 4E-BP1 or 4E-BP2 results in differential HSV1-dICP0 infectivity in the presence of asTORi. Notably, forced expression of eIF4E was recently reported to strengthen the potency of oncolytic measles virus [42]. Since wild type HSV1 infection is known to stimulate mTORC1-dependent 4E-BP1 inactivation [25], our results suggest that dysregulated eIF4E/4E-BP ratio and/or hyperactivated mTORC1 in cancer cells contribute to sustain, and ultimately favor viral protein synthesis in the presence of asTORi over antiviral mRNA translation. This model is consistent with previous data showing translation of antiviral *Irf7* mRNA to be highly sensitive to 4E-BPs [14–16], and to asTORi treatment as presented here. Nonetheless, it is noteworthy that overexpression or silencing of eIF4E in cells can induce a compensatory response vis-a-vis 4E-BP1/2 expression or 4E-BP1 phosphorylation [23, 43]. We did observe changes in expression and phosphorylation of 4E-BP1 upon altering the levels of eIF4E in the transduced cells treated with asTORi. This compensatory mechanism may have impacted partly on the replication of HSV1-dICP0.

Recently, short-term fasting was shown to enhance oncolytic HSV1 replication in glioblastoma cell lines and tumours; critically, this was not observed in normal astrocytes [39]. 4E-BP1 phosphorylation was sustained during fasting in the glioblastoma cell lines, which was not the case in normal astrocytes [39], highlighting a resistance to mTORC1 inhibition and translation repression phenotype in transformed cells, similar to that described here. High eIF4E versus 4E-BP expression is suggested as a cancer prognosis measure [44], and we have shown that tumour samples of patients with hepatocellular carcinoma exhibit variable eIF4E/4E-BP1 ratios [24]. Thus, it is plausible that certain types of tumours could be selectively targeted by the pharmacoviral approach of dual mTORC1/2 inhibition and oncolytic HSV1. In addition, our results suggest that alterations in the mRNA translation machinery, in the context of elevated eIF4E or decreased 4E-BP expression, can promote HSV1 infection in the presence of asTORi (**S6 Fig**).

In our study we used an ICP0-deleted HSV1 that has been investigated and developed as an oncolytic virus platform but that has not been translated to the clinic [3, 45–47]. A limitation of our study is that the oncolytic HSV1 platforms that have progressed to clinical trials are attenuated through gamma34.5 (g34.5) gene deletion. Using the clinically relevant g34.5-deleted HSV1-1716 [34], we have observed a similar augmentation of viral infection by asTORi treatment in the transformed NT2196 cell line. Nonetheless, additional experiments with g34.5-deleted HSV1 are necessary to fully address the clinical implications of the findings presented here. Furthermore, oncolytic HSV1 research is now focused on improving immunotherapeutic anti-tumour responses, and the virus is administered together with checkpoint inhibitors in clinical trials [48]. Finding the right balance of immunosuppression during delivery of the oncolytic virus (to minimize innate antiviral responses) while retaining the humoral immunity required for an efficient and durable anti-tumour response will prove challenging. Indeed, “pre-conditioning” with the immunosuppressive drug cyclophosphamide during viral delivery has been shown to increase viral loads in several pre-clinical studies and early-phase trials [49]. However, this regimen may not elicit potent anti-tumour immune responses. Another concern is that asTORi treatment can reactivate latent HSV1 infections, the sequelae of which could complicate patient outcomes. This has been observed in a latently infected neuron culture model [50] as well as in organ transplant patients treated with immunosuppressive agents including mTOR inhibitors [51]. Despite these concerns, augmenting the initial phase of viral replication specifically within tumour tissues, either through transient systemic or localized intra-tumoural mTOR inhibitor therapy, could provide the added benefit of reducing cancer cell proliferation and inducing a subsequent increase in viral replication, which upon drug removal could elicit a more potent therapeutic immune response [52]. Such transient or tumour-targeted mTOR inhibition would need to be investigated in additional immune-competent cancer animal models to determine the correct dosing and its potential therapeutic efficacy in combination with oncolytic HSV1.

Finally, other complementary pathways (e.g. MEK/ERK and MNK/eIF4E phosphorylation) can become activated during prolonged mTOR inhibition or upon infection by viruses [21, 53, 54]. While we did not assess the contribution of these alternative signaling cascades here, additional studies are required to investigate their functions in cellular and HSV1 mRNA translational control during mTOR inhibition. In conclusion, we have shown that asTORi treatment induces a cellular state in transformed cell lines or cells exhibiting a dysregulated eIF4E/4E-BPs axis that HSV1 usurps for increased replication.

## Materials and methods

### Cell lines, viruses, and mTOR inhibitors

The human cell lines: foreskin fibroblast HFF, transformed embryonic kidney HEK293T, malignant glioblastomas U251N and HTB-14, hepatocellular carcinoma Huh7, the non-transformed neuroblasts SHEP, and colon carcinoma HCT116 were obtained from ATCC and cultured in Dulbecco's modified Eagle's medium (DMEM-Wisent) supplemented with 10% fetal bovine serum (Wisent) and 100 units/ml Penicillin-Streptomycin (Wisent). The transformed murine mammary gland cell line 4T1 (ATCC) was propagated in Roswell Park Memorial Institute medium 1640 (RPMI1640-Wisent) supplemented with 10% fetal bovine serum and 100 units/ml Penicillin-Streptomycin. The normal murine mammary gland cells NMuMG and the NeuT transformed NMuMG, NT2196, described in [33] were generously provided by Dr. William J. Muller. Both cell lines were cultured using the same DMEM medium as before but with the addition of 10mM HEPES pH7.5 (Wisent) and 10μg/ml insulin (Wisent). NT2196 medium was also supplemented with 1μg/ml puromycin (Bioshop). The mTOR inhibitors Rapamycin, PP242, and Torin1 were purchased from Sigma, INK1341 and INK128 were obtained from Intellikine INC, as previously described in [23, 55]. TRCN0000468751 (pLX317 EIF4EBP1) and TRCN0000471416 (pLX317 eIF4E) lentiviral vectors from the MISSION TRC3 Human LentiORF Collection (Sigma) were used to generate cells stably over-expressing wild type human eIF4E or 4E-BP1. 4E-BP1/2 and eIF4E shRNA vectors were previously described [23]. Wild type Herpes simplex virus 1 (HSV1) KOS strain, GFP-expressing HSV1-dICP0 mutant and FLuc-expressing HSV1-dICP0 have been previously described [3]. The wild type HSV1 and HSV1-dICP0 were propagated and titered on Vero cells, following purification and concentration via sucrose cushion ultracentrifugation. The GFP-expressing HSV1-1716 was provided by Virttu Biologics, UK / Sorrento Therapeutics, San Diego, USA.

### Western blot analysis

Cells were washed with ice-cold PBS and lysed on ice using RIPA buffer (50mM Tris-HCl pH 8.0, 150mM NaCl, 0.1% SDS, 0.5% Sodium deoxycholate, 1% Triton X100, 10mM Sodium Fluoride, 1mM Sodium orthovanadate, 1mM DTT and protease inhibitor cocktail (Roche)). Cell debris were removed by centrifugation at 10,000g for 10min at 4°C. Protein concentration was determined using BioRad assay. Herpes Simplex Virus Type 1 polyclonal antibody was purchased from Dako (Agilent Technologies), total 4E-BP1, 4E-BP2, phospho-S240–S244 rpS6 antibodies were from cell signaling, total rpS6 and alpha-tubulin antibodies were from Santa Cruz, eIF4E antibody was from BD Biosciences, β-actin antibody was from Sigma and V5-tag antibody was from Life Technologies. Anti-4E-BP3 number 1791 was previously described [20].

### ISRE-promoter and HEKBLUE (human) type-I IFN assay

The plasmids encoding the ISRE promoter linked to firefly luciferase and Renilla luciferase (transfection control) were transfected using Lipofectamine and Plus reagent (Invitrogen) according to the manufacturer’s instruction. Cell extracts were prepared in 1X passive lysis buffer (Promega) 48 hours post-transfection and assayed for RLuc and FLuc activity in a Lumat LB95507 bio luminometer (EG and G Bertold) using a dual-luciferase reporter assay system (Promega) according to the manufacturer’s instructions. FLuc activity was normalized against RLuc activity. For the HEKBLUE assay, cells were transfected with 1μg/ml poly(I:C) (InvivoGen) using lipofectamine 2000 (Life Technologies) as per manufacturer’s instructions. Type-I IFN-containing supernatant was collected 24 hours post-transfection. HEKBLUE cells were seeded into a 96well plate (50,000 cells per well), mixed with 20μl of supernatant for a total volume of 200μl per well and incubated overnight to allow for the expression of Secreted Embryonic Alkaline Phosphatase (SEAP). To quantify the levels of IFN-induced SEAP, 160 μl of Quanti-Blue (InvivoGen) were mixed with 40μl of supernatant and incubated at 37°C for 30min. The absorbance of the resulting reaction was measured at 650nm.

### Cell viability and live cell monitoring of virus spread

NMuMG, NT2196 and 4T1 cells were infected at the indicated multiplicity of infection for 96 hours after which cells were fixed using 20% TCA solution for 5min and stained with crystal violet for 30 min followed by gentle wash with distilled water. Crystal violet stock solution was prepared by dissolving 1g of crystal violet in 100ml of 20% ethanol. Working solution prepared by mixing 20ml of the stock solution with 40ml of 100% ethanol and 140ml of distilled water. For quantification purposes, remaining viable cells from a separate experiment were manually counted using a hemacytometer and Trypan Blue exclusion. Live cell monitoring of virus spread was measured using the IncuCyte ZOOM system (Essen BioScience, MI, US). NMuMG and NT2196 cells were seeded at 80–90% confluency and were pretreated with DMSO or PP242 prior to infection at the indicated MOI. Multiple images per well were taken every 2 hours until the predermined end point. Images were then analyzed using the IncuCyte ZOOM software (Essen BioScience, MI, US) to calculate for the GFP cluster integrated intensity (Green calibrated unit x μm^2^) as a measurement of virus infection.

### *In Vivo* assessment of PP242/HSV1-dICP0 combination

In order to assess the *in vivo* efficacy of PP242/HSV1 combination, 3x10^5^ 4T1 cells were injected subcutaneously in the right flank of syngeneic mice. PP242 or vehicle was administered by gavage at 60mg/kg at 10, 12, 14 and 17 days post-implantation. FLuc expressing HSV1-dICP0 was intra-tumourally injected at 10 million pfu on days 11, 13 and 18. HSV1-dICP0 spread was assessed by measuring luciferase activity using IVIS at day 14. Tumour size was monitored using electronic caliper. End point at which animals were sacrificed was set to tumour size 15mm X 15mm.

### RNA extraction, quality control, reverse transcription, semi-quantitative and qPCR

Adherent cells were lysed using Trizol reagent (Life Technologies) immediately in the plate. RNA was isolated as per manufacturer’s instructions. RNA quality control and qPCR methodology was adopted from “the Minimum Information for Publication of Quantitative Real-Time PCR Experiments” (MIQE) guidelines [56, 57]. RNA purity was verified by nanodrop and A260/A280 ratio for all samples used was between 1.8 and 2. RNA integrity was verified by denaturing TBE/agarose gels. An equal amount of total RNA per condition for IFNβ level assessment following HSV1 stimulation were reverse transcribed into cDNA using SuperScript III Reverse Transcriptase (Invitrogen) following manufacturer’s instructions. Random Hexamer, RiboLock ribonuclease inhibitor and dNTPs were purchased from Thermo Scientific. Transcripts abundance was determined by qPCR (eppendorf realplex 2) using iQ SYBR Green Supermix from Bio-Rad following manufacturer’s instructions. qPCR and sqPCR primers were designed using the NCBI primer blast software (https://www.ncbi.nlm.nih.gov/tools/primer-blast/) following MIQE guidelines for optimal primer length, GC content and Tm.

List of primers:

HSV1 gC Fwd: 5’-GCCAGATCGACACGCAGACG-3’

HSV1 gC Rev: 5’-CGAAATGGGCAGGGTGGACC-3’

HSV1 ICP4 Fwd: 5’-CGACACGGATCCACGACCC-3’

HSV1 ICP4 Rev: 5’-ATCCCCCTCCCGCGCTTCGTCCG-3’

HSV1 TK qPCR Fwd: 5’- TCGGGGACACGTTATTTACCCTG-3’

HSV1 TK qPCR Rev: 5’- GCCCAGGCAAACACGTTATACAG-3’

hβ-Actin Fwd: 5’-GGACTCCTATGTGGGTGACGAGG-3’

hβ-Actin Rev: 5’ -GGGAGAGCATAGCCCTCGTAGAT-3’

mβ-Actin qPCR Fwd: 5’-GTACCACCATGTACCCAGGC-3’

mβ-Actin qPCR Rev: 5’-CGCAGCTCAGTAACAGTCCG-3’

hIFNβ Fwd: 5’-AATTGAATGGGAGGCTTGAA-3’

hIFNβ Rev: 5’-AGCCAGGAGGTTCTCAACAA-3’

mIFNβ qPCR Fwd: 5’-CTCCAGCACTGGGTGGAATG-3’

mIFNβ qPCR Rev: 5’-AGTGGAGAGCAGTTGAGGAC-3’

mISG15 qPCR Fwd: 5’- TGGTACAGAACTGCAGCGAG-3’

mISG15 qPCR Rev: 5’- AGCCAGAACTGGTCTTCGTG-3’

mIRF7 qPCR Fwd: 5’- GCACTTTCTTCCGAGAACTGGAGG-3’

mIRF7 qPCR Rev: 5’- GTCTTGCCCAAAACCCAGGTA-3’

Fluc qPCR Fwd: 5’- ATCCGGAAGCGACCAACGCC-3’

Fluc qPCR Rev: 5’- GTCGGGAAGACCTGCCACGC-3’

### Metabolic radiolabeling

Cells cultured in complete growth media were incubated for 30 min at 37°C with complete culture medium containing 10 μCi/ml [^35^S]methionine. After 30 min incubation, cells were washed with PBS twice and lysed in Laemmli sample buffer. Incorporation was measured by TCA precipitation of proteins followed by scintillation counting. Radiolabeled proteins were also separated by SDS-PAGE followed by autoradiography.

### Polysome profile analysis

Sucrose density gradients (10 to 50%) were prepared using Biocomp Gradient Master 108 as per manufacturer’s instructions and as previously published [58]. 10% and 50% sucrose solutions were prepared in a buffer containing 20 mM HEPES-KOH pH 7.6, 100 mM KCl, 5 mM MgCl_2_, 100 μg/ml cycloheximide, EDTA-free protease inhibitors mixture tablets (Roche), and 200 units/ml ribonuclease inhibitor (Ribolock from Thermo Fisher Scientific). A total of 7.5x10^6^ cells for 4T1 and NMuMG and 15x10^6^ for NT2196 were seeded per 15cm dish 24 hours prior to HSV1-dICP0 infection. Cells were pretreated for 30min with vehicle or PP242 followed by infection with GFP-expressing HSV1-dICP0 at 0.1 MOI for 24 hours. Prior to collection, cells were treated with 100μg/ml cycloheximide for 5 min at 37°C, washed twice with ice-cold PBS containing 100 μg/ml cycloheximide and collected by scraping. Cell pellet following centrifugation at 1000 rpm for 10min at 4°C were lysed in hypotonic lysis buffer (5 mM Tris pH 7.5, 2.5 mM MgCl_2_, 1.5 mM KCl, EDTA-free protease inhibitors cocktail tablets (Roche), 100 μg/ml cycloheximide, 2 mM DTT, 200 units/ml Ribolock, 0.5% (v/v) Triton X-100, and 0.5% (w/v) sodium deoxycholate. Cell debris were cleared by centrifugation at 21,000g for 5 min at 4°C. RNA concentration was determined using a Thermo Scientific Nanodrop 2000 spectrophotometer. A total of 500 μg total RNA per condition were loaded on each gradient, which were spun at 36,000 rpm (230501 X g) for 2 hours at 4°C using a Beckman coulter SW40Ti rotor and Optima L80 XP ultracentrifuge. Gradients were fractionated using Teledyne ISCO fractionator with the pump set at 1.5ml per min. Optical density at 254nm was recorded at 10 measurements per second frequency. RNA from each fraction was isolated using TRIzol (Invitrogen) and treated with DNaseTurbo (Ambion) according to the manufacturer's instructions. Reverse transcription PCR (RT-PCR) and quantitative RT-PCR (qRT-PCR) reactions were carried out using SuperScript III First-Strand Synthesis System (Invitrogen) and iQ SYBR Green Supermix (BIO-RAD) according to the manufacturer's instructions. For qPCR experiments, each fraction was spiked with 5ng of polyA+ firefly luciferase mRNA (Promega) prior to extraction. Measurements were then normalized to luciferase abundance, and plotted as relative transcript amount over luciferase control.

### Preparation of reporter mRNAs and luciferase reporter assay

Luciferase reporter mRNAs were generated using MAXIscript T7 in Vitro Transcription kit (Ambion) according to the manufacturer’s protocol in the presence of the cap analog. PCR products encoding a T7 promoter followed by luciferase and a poly(A) sequence were used as templates for in vitro transcription. 4T1 cells were seeded on a 24-well-plate and cultured overnight. Cells were transfected with capped HSV-ICP0-5’UTR-Rluc-pA, HSV-TK-5’UTR-Rluc-pA, IRF7-5’UTR-Rluc-pA or Rluc-pA together with capped Fluc-pA as a transfection control using Lipofectamine (Invitrogen). Cells were treated with 1μM PP242 for 24 hours and lysed 24 hours post transfection. Luciferase activities were determined using Dual-Luciferase Reporter Assay System (Promega) according to the manufacturer's instruction.

### Statistical analyses

Statistical tests were performed within Prism (GraphPad) or Excel. Error bars for data presented are standard deviation (SD) from the mean. P values were calculated as follow: For comparison between two independent groups, a two-tailed unpaired *t* test was used. For comparison between more than two independent groups, a one-way analysis of variance was performed with Dunnett's or Bonferroni's post hoc tests, and adjusted *p* values were reported. *p* values are reported as follows: *, *p* < 0.05; **, *p* < 0.01; ***, *p* < 0.001; ns denotes non-significant *p* values.

### Ethics statement

The animal experiment was performed in accordance with the guidelines for animal care at the University of Ottawa Animal Care and Veterinary Services under the approved protocol OGHRI-58. The University of Ottawa is a registered research facility under the Animals for Research Act and is certified by the Canadian Council on Animal Care.

## Supporting information

S1 Fig asTORi treatment enhances HSV1-dICP0 and g34.5-deleted HSV1-1716 infection but limits the propagation of other oncolytic viruses. (A-C) U251N cells were pretreated with DMSO (control), PP242 (2μM) or rapamycin (RAP 100nM) for 30 min and infected with GFP-expressing HSV1-dICP0, GFP-expressing myxoma virus, or GFP-expressing VSV^Δ51M^ at a MOI of 0.1 in the presence of the inhibitors. Relative infection was monitored at different time points post-infection by fluorescence microscopy (GFP-expressing HSV1-dICP0 (A) and GFP-expressing myxoma virus (B)), or by Western blot (VSV^Δ51M^ (C)). (D) Mouse mammary carcinoma cell line 4T1 was pretreated with DMSO, rapamycin (100nM) or PP242 (2μM) for 30 min and infected with GFP-expressing HSV1-dICP0, GFP-expressing myxoma virus, GFP-expressing vaccinia virus JX594, or GFP-expressing VSV^Δ51M^ at a MOI of 0.1 in the presence of the inhibitors. Relative infection was monitored 48 hours post-infection by fluorescence microscopy. (E) The transformed NT2196 and non-transformed NMuMG cells were pretreated with DMSO or PP242 (2μM) for 30 min followed by infection with GFP-expressing HSV1-dICP0 (left) or GFP-expressing g34.5-deleted HSV1-1716 (right), both viruses at a MOI of 0.1. GFP fluorescence units measured using IncuCyte Zoom every 2 hours over a period of 48 hours are presented. Fluorescent and brightfield pictures are also included. (F) HSV1-dICP0 titers at 48 hours post-infection obtained from the transformed 4T1 and NT2196 and the non-transformed NMuMG cells when pretreated with DMSO, rapamycin (100nM), PP242 (2μM), or INK1341 (100nM). Results are presented as titers normalized to DMSO control set at 100% ± SD (n = 3)).
(TIF)

S2 Fig HSV1-dICP0 is potentiated in cancer cell lines by different asTORi. (A) Transformed human cell lines HEK293T and HCT116 were pretreated with DMSO, PP242 (2μM), INK1341 (100nM), or rapamycin (RAP 100nM) for 30 min and infected with GFP-expressing HSV1-dICP0 at a MOI of 0.1 for 48 hours in the presence of the inhibitors. Viral protein expression was monitored by Western blot using antibodies against HSV1 antigens; drug efficacy was monitored by phosphorylation of rpS6 and 4E-BP1. Total rpS6 and β-actin expression were used as loading controls. (B) Huh7 malignant hepatocellular carcinoma cells were pretreated with DMSO, PP242 (2μM) or INK1341 (100nM) for 30 min and infected with GFP-expressing HSV1-dICP0 at a MOI of 0.1 in presence of the inhibitors. Cell oncolysis was monitored by crystal violet staining of live cells 72 hours post-infection (C) Transformed 4T1 and NT2196, and non-transformed NMuMG cells were infected with GFP-expressing HSV1-dICP0 in the presence of DMSO, PP242 (2μM), INK128 (100nM), or Torin1 (100nM), pretreated for 30 min prior to infection. In this particular experiment, 4T1 and NT2196 cells were infected at a MOI of 0.1 while the NMuMG cells were infected at a MOI of 1. Virus infection was assessed 48 hours post-infection by fluorescence microscopy. (D) Non-transformed cell lines SHEP and NMuMG were pretreated as in (A) and infected with GFP-expressing HSV1-dICP0 at a MOI of 0.1 for 48 hours. Viral protein expression was monitored by Western blot. (E) ImageJ quantification of the percentage of GFP positive cells following infection of 4T1, NMuMG or NT2196 in presence of DMSO, PP242 (2μM) or INK1341 (100nM). Results are presented as total percentage of GFP positive cells ± SD (n = 3).
(TIF)

S3 Fig asTORi treatment reduces HSV1-induced type-I IFN responses in normal and cancer cells. (A) Non-transformed mouse embryonic fibroblasts (MEFs) or the human glioma cell line U251N were infected with wild type HSV1 in the presence of DMSO, rapamycin (100nM) or PP242 (2μM). *Ifn-β* mRNA levels were measured 24 hours post-infection by RT-PCR. (B) Graphical representation of type-I IFN protection assay shown in Fig 3C: Type-I IFN production was induced by transfecting cells with poly(I:C) RNA in the presence of DMSO, rapamycin, or PP242, and incubated overnight. The supernatant containing secreted type-I IFN was used to condition naïve cells for 6 hours followed by wild type HSV1 infection. Infected cells were lysed 24 hours post-infection for analysis by Western blot and virus titration. (C) HEKBLUE assays performed on normal HFF cell line and glioblastoma cell lines U343 and U373 treated for 6 hours with poly(I:C) in presence of DMSO, Rapamycin (RAP 100nM), PP242 (2μM), or Torin1 (100nM). Quanti BLUE type I IFN detection was assessed by the levels of secreted alkaline phosphatase and measure by OD at 650nM. UV absorbance profiles (254nm) of ribosomes isolated from 4T1 cells (D) and NT2196 cells (F) pretreated with DMSO or PP242 (2μM) for 30 min prior to infection with HSV1-dICP0 at a MOI of 0.1 for 24 hours. 40S, 60S, and 80S denote the corresponding ribosomal subunits and monosomes, respectively. Western blotting performed at 48 hours during the same experiment showing an increase in HSV1-dICP0 protein synthesis. (E) Total amount and polysome distribution of *β-actin*, *Irf7*, *gC* and *ICP4* mRNAs from DMSO- or PP242-treated and infected 4T1 cells was determined by semi-quantitative RT-PCR (sqRT-PCR). (G) Polysome distribution of *β-actin*, *Irf7*, *Isg15* and *TK* mRNAs from the fractions of DMSO- or PP242-treated and infected NT2196 cells was determined by quantitative RT-PCR (qRT-PCR) and presented as relative transcript amount in each fraction normalized to spiked luciferase mRNA control. (H) Luciferase reporter constructs containing the 5’ UTR of *ICP0*, *TK*, or *Irf7* were in vitro transcribed and transfected into 4T1 cells. Renilla luciferase over Firefly luciferase was measured by Lumiglo and presented as % PP242 treated over DMSO samples.
(TIF)

S4 Fig eIF4E/4E-BP expression and the effect of asTORi on HSV1-dICP0 infection. (A) RT-qPCR measurements of the levels of immediate-early HSV1 gene ICP4 at 8 hours post-infection with HSV1-dICP0 at 0.1MOI in 4T1, NMuMG and NT2196 cell lines pretreated with DMSO or INK1341 (100nM) for 30 min prior to infection. Results are presented as total transcript levels normalized to DMSO control set to 1 ± SD (n = 3). (B) Global protein synthesis was assessed by a 30 min pulse [^35^S]methionine incorporation into newly synthesized proteins at 24 hours post-infection with wild-type HSV1 at a MOI of 1. Proteins were separated on SDS-PAGE and changes in protein synthesis revealed by autoradiography (left panels). Corresponding Western blot for HSV1 proteins in infected cells treated or not with mTOR inhibitors (right panels). (C) 786–0, 4T1 and NT2196 cells were exposed to elevated concentrations of the asTORi PP242 or INK1341, and infected with GFP-expressing HSV1-dICP0 at a MOI of 0.1 for 48 hours. Resulting virus infection was assessed by fluorescence microscopy. (D) Normal murine mammary epithelial cell line NMuMG and NT2196 transformed mammary cells were treated with increasing concentrations of the asTORi PP242 and infected with HSV1-dICP0 at a MOI of 0.1. Cell lysates were prepared at 48 hours post-infection and assessed by Western Blotting for HSV1, p-4E-BP1 (T37/46; S65; T70), total 4E-BP1 and β-actin (loading control). Note that different exposure time between NMuMG and NT2196 are presented for the HSV1 blot to demonstrate the repression of HSV1-dICP0 in NMuMG cells, versus the augmentation of HSV1-dICP0 protein expression in NT2196 cells. (E) Transformed 4T1 and NT2196 and non-transformed NMuMG cells were pretreated with PP242 (2μM) for 30 min and infected with HSV1-dICP0 at 0.1 MOI in the presence of the inhibitor. At 48 hours post-infection, viral proteins, and eIF4E and 4E-BP1/2/3 protein levels were monitored by Western Blot. β-actin expression was used as loading control. (F) Transformed 4T1 and NT2196 and non-transformed NMuMG were transduced to stably express shRNA against 4E-BP2, or scrambled shRNA control. Transduced cells were infected as above with GFP-expressing HSV1-dICP0 at 0.1 MOI in the presence of DMSO, PP242 (2μM), or INK1341 (100nM). At 48 hours post-infection, GFP fluorescence was monitored by fluorescence microscopy.
(TIF)

S5 Fig asTORi effect on HSV1 is dependent on eIF4E/4E-BP expression. (A-C) Non-transformed NIH3T3 and SHEP cells, and (D-H) transformed U251N glioma cell line were transduced to stably overexpress eIF4E, 4E-BP1, or control empty pBabe vector (A-C, F-H), or to stably express shRNA against eIF4E or scrambled control (D,E). Transduced cells were infected with GFP-expressing HSV1-dICP0 at 0.1 MOI in the presence of DMSO, PP242 (2μM) or INK1341 (100nM), pretreated for 30 min prior to infection. HSV1 proteins were assessed by Western blot (A-D, F-G), and viral infection was monitored by fluorescence microscopy (E,H).
(TIF)

S6 Fig Schema of HSV1-dICP0 infection of cells in presence of active-site mTOR inhibitors (asTORi). asTORi treatment results in a strong decrease in antiviral gene transcription and translation, but HSV1-dICP0 viral protein synthesis differs: Depicted on the left, normal cells with homeostatic eIF4E/4E-BP expression, or cells with either reduced eIF4E or elevated 4E-BP1 expression, asTORi treatment potently limits viral and host protein synthesis, resulting in limited infection and spread of the virus. Depicted in the middle, in the absence of asTORi, cellular antiviral gene transcription and translation is normally induced and controls HSV1-dICP0 propagation. Finally, depicted on the right, elevated eIF4E expression or loss of 4E-BPs in cancer cells, or cells genetically modified to overexpress eIF4E or silence 4E-BP1/2, sustain sufficient protein synthesis levels in presence of asTORi to favor HSV1-dICP0 mRNA translation while the antiviral response is limited.
(TIF)
