# Supplementary figures and images for: Active-site mTOR inhibitors augment HSV1-dICP0 infection in cancer cells via dysregulated eIF4E/4E-BP axis
Source: PLoS Pathog. 2018 Aug 23;14(8):e1007264. doi: 10.1371/journal.ppat.1007264 (PMC6124814; doi:10.1371/journal.ppat.1007264)

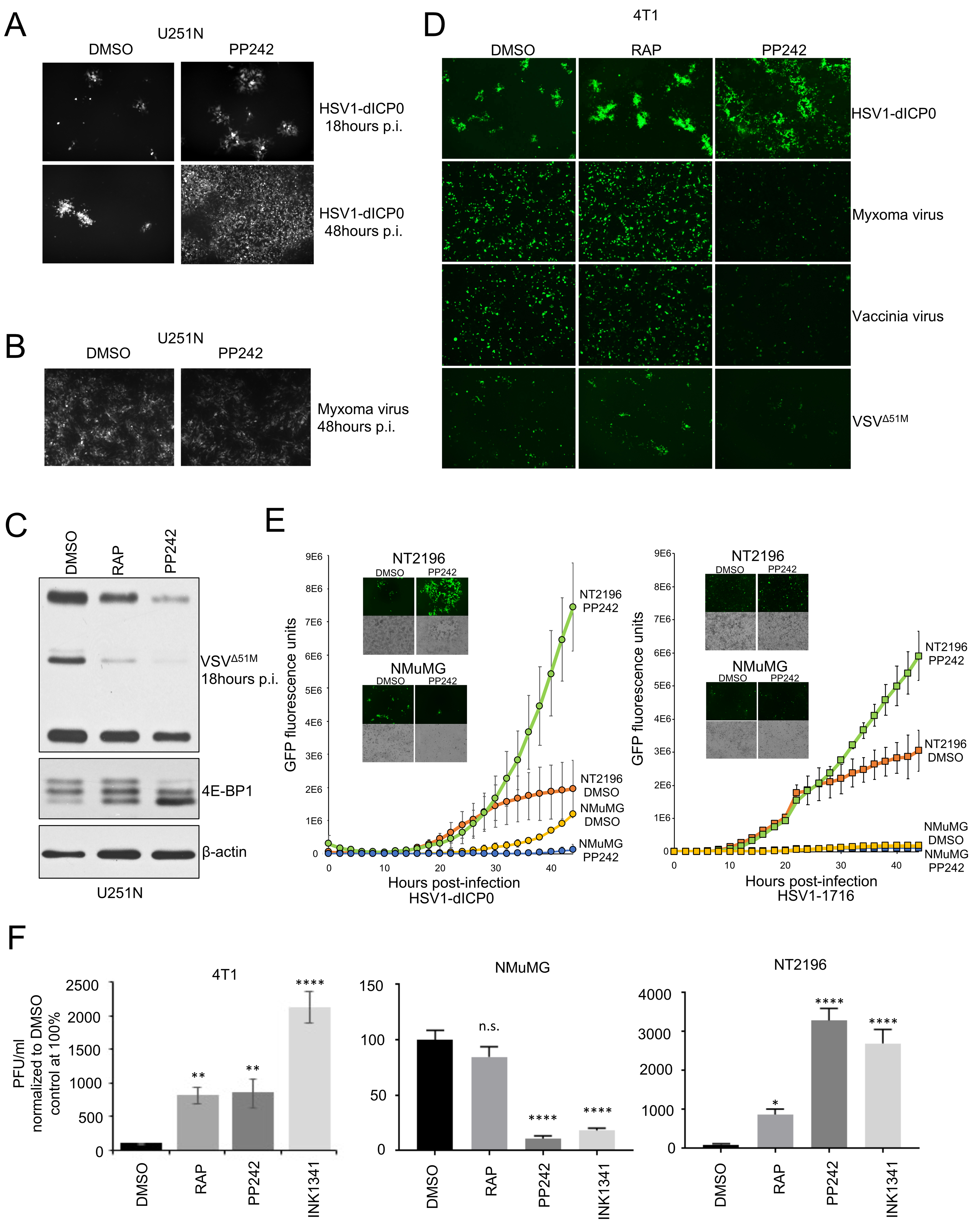

Supplement: S1 Fig — (A-C) U251N cells were pretreated with DMSO (control), PP242 (2μM) or rapamycin (RAP 100nM) for 30 min and infected with GFP-expressing HSV1-dICP0, GFP-expressing myxoma virus, or GFP-expressing VSVΔ51M at a MOI of 0.1 in the presence of the inhibitors. Relative infection was monitored at different time points post-infection by fluorescence microscopy (GFP-expressing HSV1-dICP0 (A) and GFP-expressing myxoma virus (B)), or by Western blot (VSVΔ51M (C)). (D) Mouse mammary carcinoma cell line 4T1 was pretreated with DMSO, rapamycin (100nM) or PP242 (2μM) for 30 min and infected with GFP-expressing HSV1-dICP0, GFP-expressing myxoma virus, GFP-expressing vaccinia virus JX594, or GFP-expressing VSVΔ51M at a MOI of 0.1 in the presence of the inhibitors. Relative infection was monitored 48 hours post-infection by fluorescence microscopy. (E) The transformed NT2196 and non-transformed NMuMG cells were pretreated with DMSO or PP242 (2μM) for 30 min followed by infection with GFP-expressing HSV1-dICP0 (left) or GFP-expressing g34.5-deleted HSV1-1716 (right), both viruses at a MOI of 0.1. GFP fluorescence units measured using IncuCyte Zoom every 2 hours over a period of 48 hours are presented. Fluorescent and brightfield pictures are also included. (F) HSV1-dICP0 titers at 48 hours post-infection obtained from the transformed 4T1 and NT2196 and the non-transformed NMuMG cells when pretreated with DMSO, rapamycin (100nM), PP242 (2μM), or INK1341 (100nM). Results are presented as titers normalized to DMSO control set at 100% ± SD (n = 3)). (TIF) [file ppat.1007264.s001.tif]

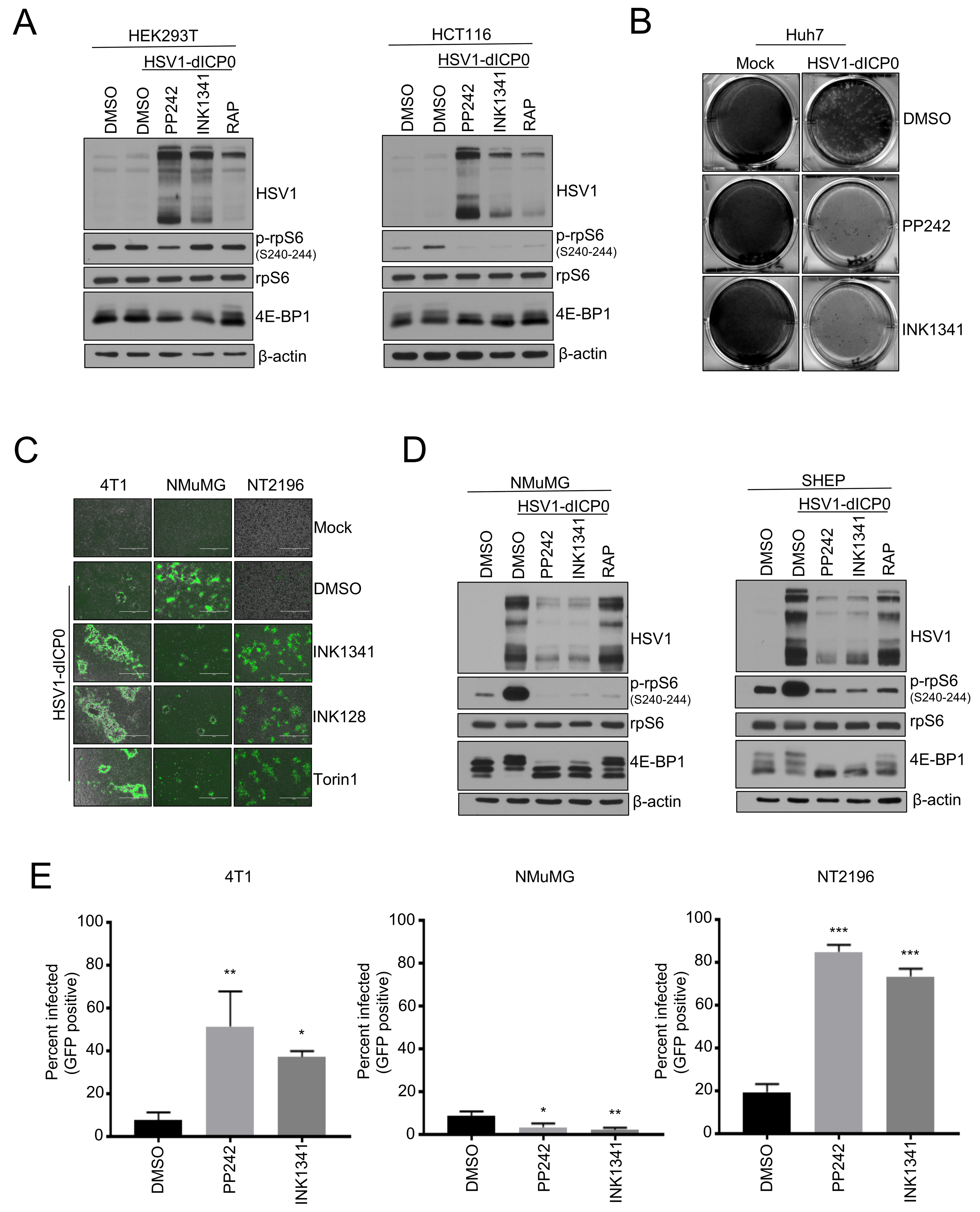

Supplement: S2 Fig — (A) Transformed human cell lines HEK293T and HCT116 were pretreated with DMSO, PP242 (2μM), INK1341 (100nM), or rapamycin (RAP 100nM) for 30 min and infected with GFP-expressing HSV1-dICP0 at a MOI of 0.1 for 48 hours in the presence of the inhibitors. Viral protein expression was monitored by Western blot using antibodies against HSV1 antigens; drug efficacy was monitored by phosphorylation of rpS6 and 4E-BP1. Total rpS6 and β-actin expression were used as loading controls. (B) Huh7 malignant hepatocellular carcinoma cells were pretreated with DMSO, PP242 (2μM) or INK1341 (100nM) for 30 min and infected with GFP-expressing HSV1-dICP0 at a MOI of 0.1 in presence of the inhibitors. Cell oncolysis was monitored by crystal violet staining of live cells 72 hours post-infection (C) Transformed 4T1 and NT2196, and non-transformed NMuMG cells were infected with GFP-expressing HSV1-dICP0 in the presence of DMSO, PP242 (2μM), INK128 (100nM), or Torin1 (100nM), pretreated for 30 min prior to infection. In this particular experiment, 4T1 and NT2196 cells were infected at a MOI of 0.1 while the NMuMG cells were infected at a MOI of 1. Virus infection was assessed 48 hours post-infection by fluorescence microscopy. (D) Non-transformed cell lines SHEP and NMuMG were pretreated as in (A) and infected with GFP-expressing HSV1-dICP0 at a MOI of 0.1 for 48 hours. Viral protein expression was monitored by Western blot. (E) ImageJ quantification of the percentage of GFP positive cells following infection of 4T1, NMuMG or NT2196 in presence of DMSO, PP242 (2μM) or INK1341 (100nM). Results are presented as total percentage of GFP positive cells ± SD (n = 3). (TIF) [file ppat.1007264.s002.tif]

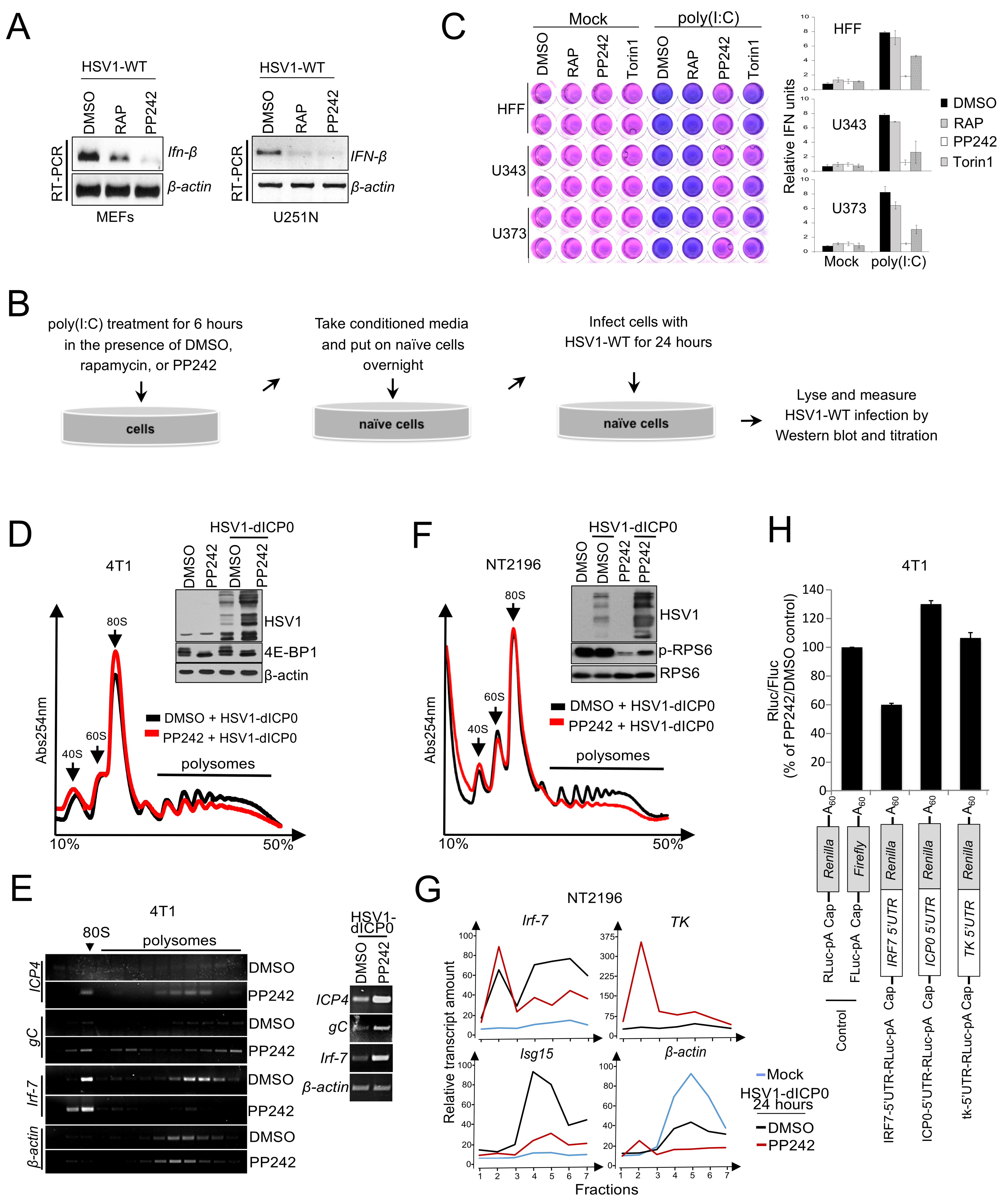

Supplement: S3 Fig — (A) Non-transformed mouse embryonic fibroblasts (MEFs) or the human glioma cell line U251N were infected with wild type HSV1 in the presence of DMSO, rapamycin (100nM) or PP242 (2μM). Ifn-β mRNA levels were measured 24 hours post-infection by RT-PCR. (B) Graphical representation of type-I IFN protection assay shown in Fig 3C: Type-I IFN production was induced by transfecting cells with poly(I:C) RNA in the presence of DMSO, rapamycin, or PP242, and incubated overnight. The supernatant containing secreted type-I IFN was used to condition naïve cells for 6 hours followed by wild type HSV1 infection. Infected cells were lysed 24 hours post-infection for analysis by Western blot and virus titration. (C) HEKBLUE assays performed on normal HFF cell line and glioblastoma cell lines U343 and U373 treated for 6 hours with poly(I:C) in presence of DMSO, Rapamycin (RAP 100nM), PP242 (2μM), or Torin1 (100nM). Quanti BLUE type I IFN detection was assessed by the levels of secreted alkaline phosphatase and measure by OD at 650nM. UV absorbance profiles (254nm) of ribosomes isolated from 4T1 cells (D) and NT2196 cells (F) pretreated with DMSO or PP242 (2μM) for 30 min prior to infection with HSV1-dICP0 at a MOI of 0.1 for 24 hours. 40S, 60S, and 80S denote the corresponding ribosomal subunits and monosomes, respectively. Western blotting performed at 48 hours during the same experiment showing an increase in HSV1-dICP0 protein synthesis. (E) Total amount and polysome distribution of β-actin, Irf7, gC and ICP4 mRNAs from DMSO- or PP242-treated and infected 4T1 cells was determined by semi-quantitative RT-PCR (sqRT-PCR). (G) Polysome distribution of β-actin, Irf7, Isg15 and TK mRNAs from the fractions of DMSO- or PP242-treated and infected NT2196 cells was determined by quantitative RT-PCR (qRT-PCR) and presented as relative transcript amount in each fraction normalized to spiked luciferase mRNA control. (H) Luciferase reporter constructs containing the 5’ UTR of ICP0, TK, or Irf7 w [file ppat.1007264.s003.tif]

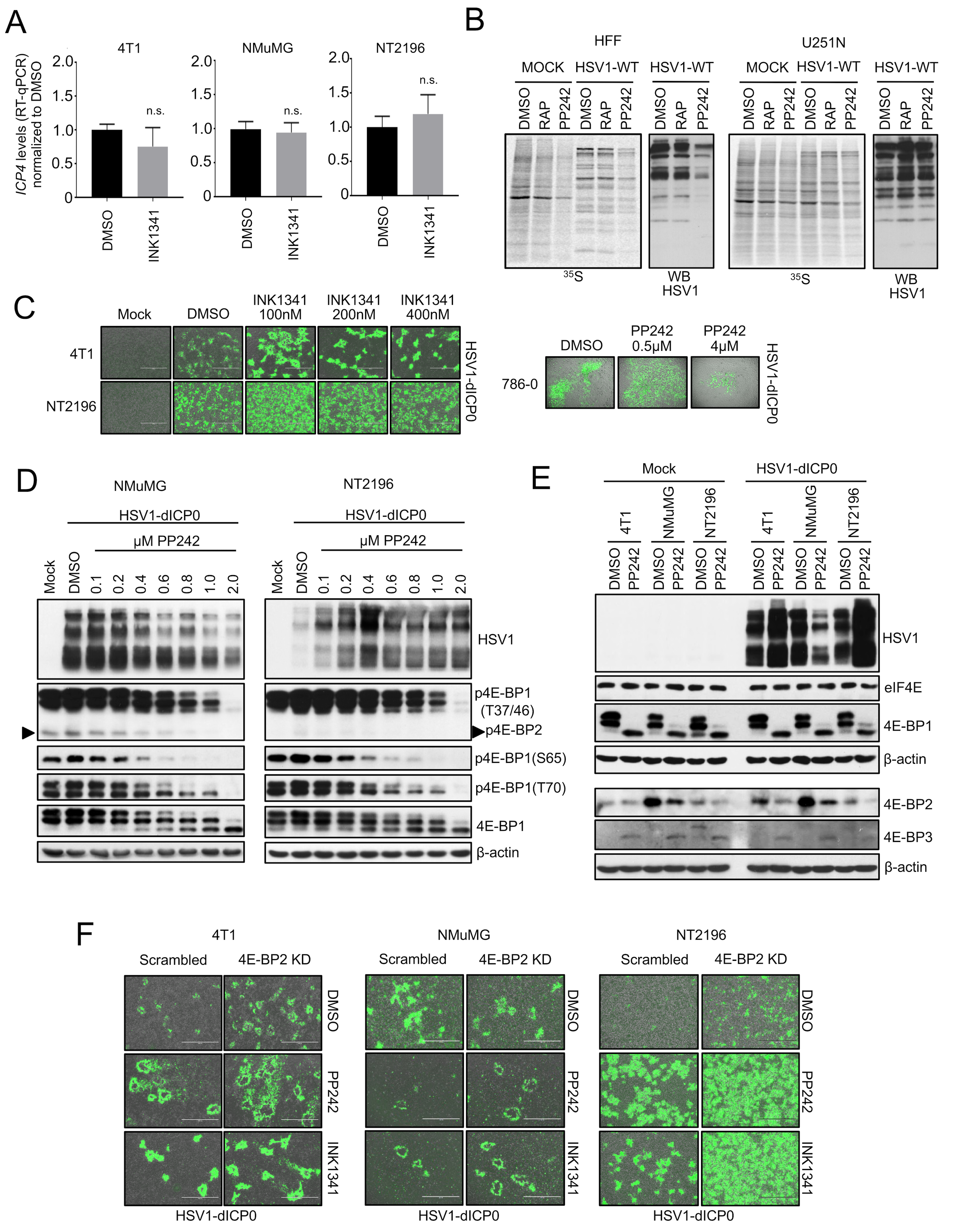

Supplement: S4 Fig — (A) RT-qPCR measurements of the levels of immediate-early HSV1 gene ICP4 at 8 hours post-infection with HSV1-dICP0 at 0.1MOI in 4T1, NMuMG and NT2196 cell lines pretreated with DMSO or INK1341 (100nM) for 30 min prior to infection. Results are presented as total transcript levels normalized to DMSO control set to 1 ± SD (n = 3). (B) Global protein synthesis was assessed by a 30 min pulse [35S]methionine incorporation into newly synthesized proteins at 24 hours post-infection with wild-type HSV1 at a MOI of 1. Proteins were separated on SDS-PAGE and changes in protein synthesis revealed by autoradiography (left panels). Corresponding Western blot for HSV1 proteins in infected cells treated or not with mTOR inhibitors (right panels). (C) 786–0, 4T1 and NT2196 cells were exposed to elevated concentrations of the asTORi PP242 or INK1341, and infected with GFP-expressing HSV1-dICP0 at a MOI of 0.1 for 48 hours. Resulting virus infection was assessed by fluorescence microscopy. (D) Normal murine mammary epithelial cell line NMuMG and NT2196 transformed mammary cells were treated with increasing concentrations of the asTORi PP242 and infected with HSV1-dICP0 at a MOI of 0.1. Cell lysates were prepared at 48 hours post-infection and assessed by Western Blotting for HSV1, p-4E-BP1 (T37/46; S65; T70), total 4E-BP1 and β-actin (loading control). Note that different exposure time between NMuMG and NT2196 are presented for the HSV1 blot to demonstrate the repression of HSV1-dICP0 in NMuMG cells, versus the augmentation of HSV1-dICP0 protein expression in NT2196 cells. (E) Transformed 4T1 and NT2196 and non-transformed NMuMG cells were pretreated with PP242 (2μM) for 30 min and infected with HSV1-dICP0 at 0.1 MOI in the presence of the inhibitor. At 48 hours post-infection, viral proteins, and eIF4E and 4E-BP1/2/3 protein levels were monitored by Western Blot. β-actin expression was used as loading control. (F) Transformed 4T1 and NT2196 and non-transformed NMuMG were transduced [file ppat.1007264.s004.tif]

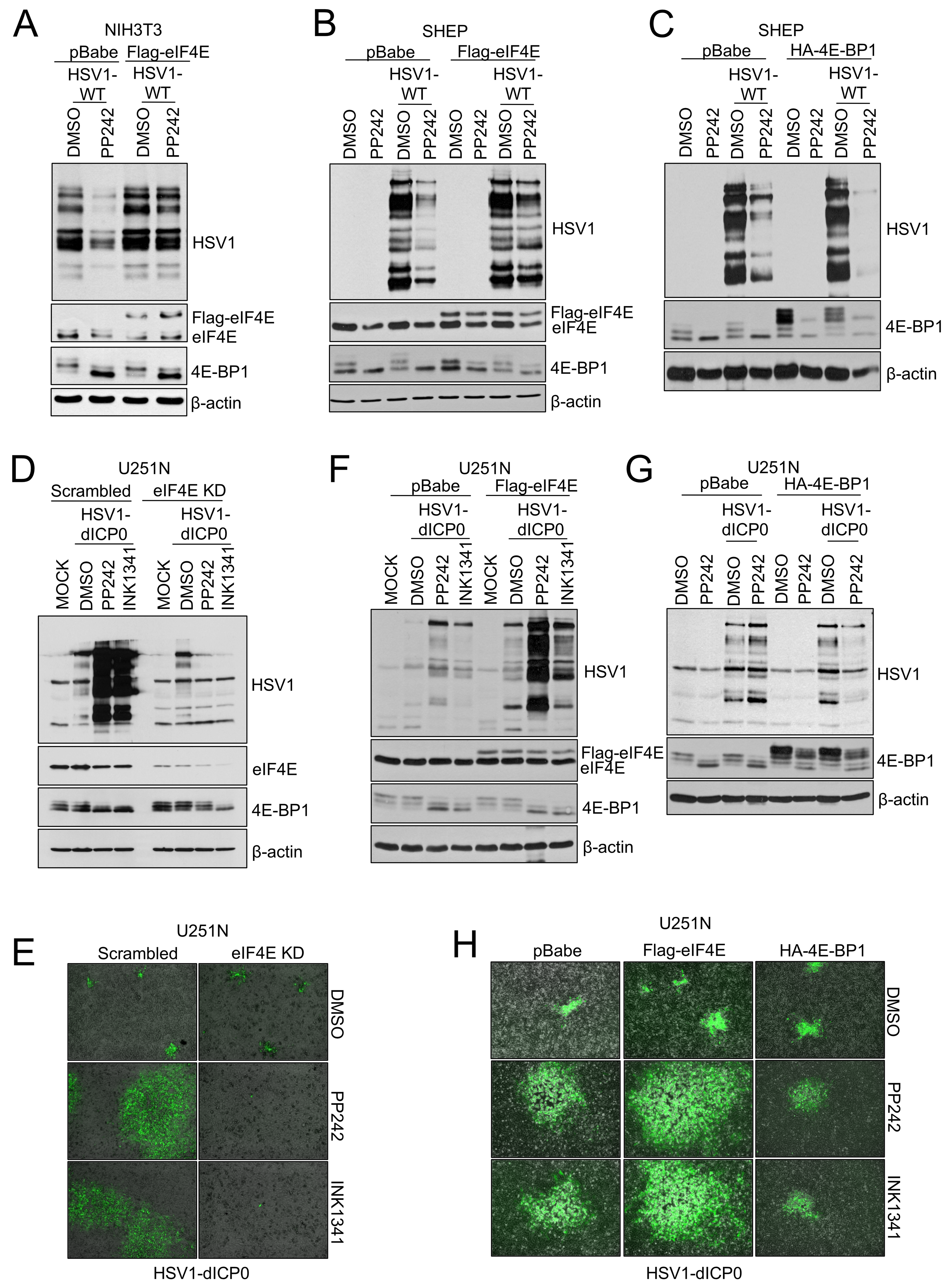

Supplement: S5 Fig — (A-C) Non-transformed NIH3T3 and SHEP cells, and (D-H) transformed U251N glioma cell line were transduced to stably overexpress eIF4E, 4E-BP1, or control empty pBabe vector (A-C, F-H), or to stably express shRNA against eIF4E or scrambled control (D,E). Transduced cells were infected with GFP-expressing HSV1-dICP0 at 0.1 MOI in the presence of DMSO, PP242 (2μM) or INK1341 (100nM), pretreated for 30 min prior to infection. HSV1 proteins were assessed by Western blot (A-D, F-G), and viral infection was monitored by fluorescence microscopy (E,H). (TIF) [file ppat.1007264.s005.tif]

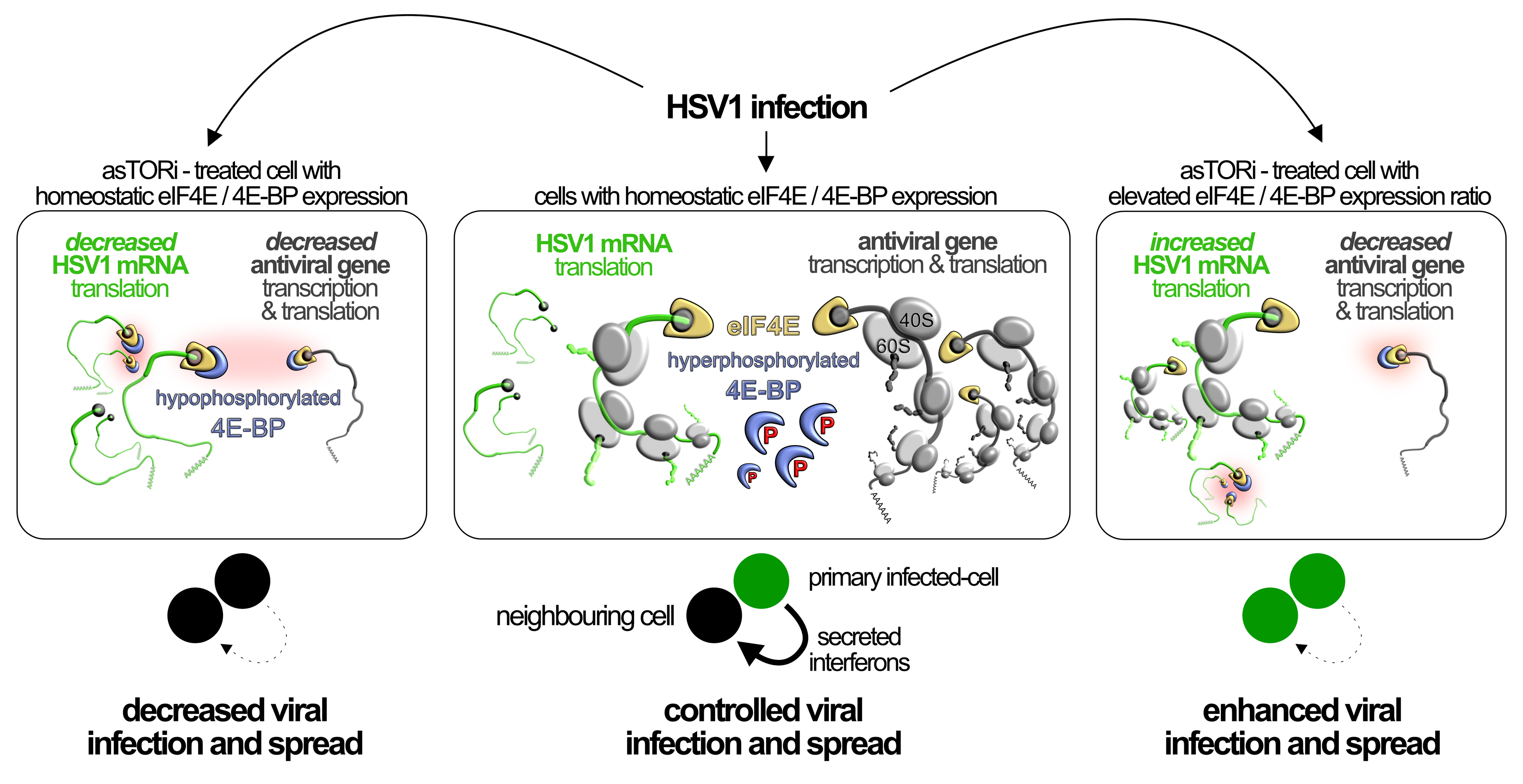

Supplement: S6 Fig — asTORi treatment results in a strong decrease in antiviral gene transcription and translation, but HSV1-dICP0 viral protein synthesis differs: Depicted on the left, normal cells with homeostatic eIF4E/4E-BP expression, or cells with either reduced eIF4E or elevated 4E-BP1 expression, asTORi treatment potently limits viral and host protein synthesis, resulting in limited infection and spread of the virus. Depicted in the middle, in the absence of asTORi, cellular antiviral gene transcription and translation is normally induced and controls HSV1-dICP0 propagation. Finally, depicted on the right, elevated eIF4E expression or loss of 4E-BPs in cancer cells, or cells genetically modified to overexpress eIF4E or silence 4E-BP1/2, sustain sufficient protein synthesis levels in presence of asTORi to favor HSV1-dICP0 mRNA translation while the antiviral response is limited. (TIF) [file ppat.1007264.s006.tif]
